# Overview of fungal terpene synthases and their regulation

**DOI:** 10.1007/s11274-023-03635-y

**Published:** 2023-05-12

**Authors:** Ricardo A. González-Hernández, Norma A. Valdez-Cruz, Martha L. Macías-Rubalcava, Mauricio A. Trujillo-Roldán

**Affiliations:** 1 Departamento de Biología Molecular y Biotecnología, Instituto de Investigaciones Biomédicas, Universidad Nacional Autónoma de México, Ciudad Universitaria, Coyoacán, C.P. 04510 Ciudad de México, México; 2 Posgrado en Ciencias Biológicas, Universidad Nacional Autónoma de México, Ciudad de México, México; 3 Departamento de Productos Naturales, Instituto de Química, Universidad Nacional Autónoma de México (UNAM), Ciudad Universitaria, Delegación Coyoacán, 04510 Ciudad de México, México

**Keywords:** Terpenes, Synthases, Regulation, Fungal cultures, Isoprene, Mevalonate pathway

## Abstract

Terpenes and terpenoids are a group of isoprene-derived molecules that constitute the largest group of natural products and secondary metabolites produced by living things, with more than 25,000 compounds reported. These compounds are synthesized by enzymes called terpene synthases, which include several families of cyclases and enzymes. These are responsible for adding functional groups to cyclized structures. Fungal terpenoids are of great interest for their pharmacological properties; therefore, understanding the mechanisms that regulate their synthesis (regulation of the mevalonate pathway, regulation of gene expression, and availability of cofactors) is essential to direct their production. For this reason, this review addresses the detailed study of the biosynthesis of fungal terpenoids and their regulation by various physiological and environmental factors.

## Biological importance of terpenes and terpenoids in fungi

Terpenes are molecules derived from isopentenyl diphosphate (IPP) and dimethylallyl diphosphate (DMAPP), which only possess carbon and hydrogen, while terpenoids originate when functional groups are added to the terpene (Christianson 2008; Palenzuela et al. 2021). The functions of these molecules in fungi include electron transport, cell wall, and membrane formation, chemical defense against predators, and establishment of symbiotic relationships, among others (Cale et al. 2016; Calvo et al. 2002; Jansen and de Groot 2004; Gershenzon & Dudareva 2007; Langenheim 1994; Weikl et al. 2016). Fungal terpenoids are of great interest due to their biological activities, such as toxins, antimicrobial, antitumor, cytotoxic, and anti-inflammatory properties (Akihisa et al. 2007; Bryant et al. 2017; Chi et al. 2018; Choi et al. 2014; El-Mekkaway et al. 1998; González et al. 2002; Han et al. 2013; Hartley et al. 2009; Hirota et al. 2002; Ishikawa et al. 2005; Li et al. 2012; Li et al. 2013; Liu et al. 2010; Mazur et al. 1996; Mothana et al. 2000; Ou et al. 2012; Schrader & Bohlmann 2015; Shibata et al. 1998; Tabuchi et al. 2020; Valdivia et al. 2005; Wang et al. 2007; Yang et al. 2017; Yin et al. 2020; Ying et al. 2013; Yue et al. 2010). Most terpenoids of pharmacological interest have been identified in basidiomycetes, while toxin production studies have been carried out in ascomycetes (Schrader & Bohlmann 2015). Due to their importance, efforts have been made to improve terpenoid production by applying OSMAC (One Strain Many Compounds) strategies in the cultivation of fungi, which have been shown to affect terpenoid synthesis, including agitation, aeration, salinity, pH, carbon, and nitrogen sources (Cui et al. 2015; Fang and Zhong 2002; Hu et al. 2017; Seo et al. 2009; Tang & Zhong 2002).

## Terpene biosynthesis in fungi

Terpenes are the largest group of secondary metabolites, generally lipophilic, and their biosynthesis in fungi depends on the levels of IPP and DMAPP. Terpenes are classified based on the number of carbons in their structure: hemiterpenes (5 carbons), monoterpenes (10 carbons), sesquiterpenes (15 carbons), diterpenes (20 carbons), sesterpenes (25 carbons), triterpenes (30 carbons). When the terpene structure presents atoms other than carbon and hydrogen, for example, oxygen, they are called terpenoids (Quin et al. 2014; Schmidt-Dannert 2015). In addition, a group of terpenoids of mixed biosynthetic origin arises from the addition of isoprenoid chains to molecules that come from another metabolic pathway. This group includes the meroterpenoids, indole terpenoids, and prenylated aromatic compounds (Christianson 2008; Nelson et al. 2005; Schrader & Bohlmann 2015), which will be addressed in this review.

## Mevalonate (MEV) pathway

**Fig. 1:**
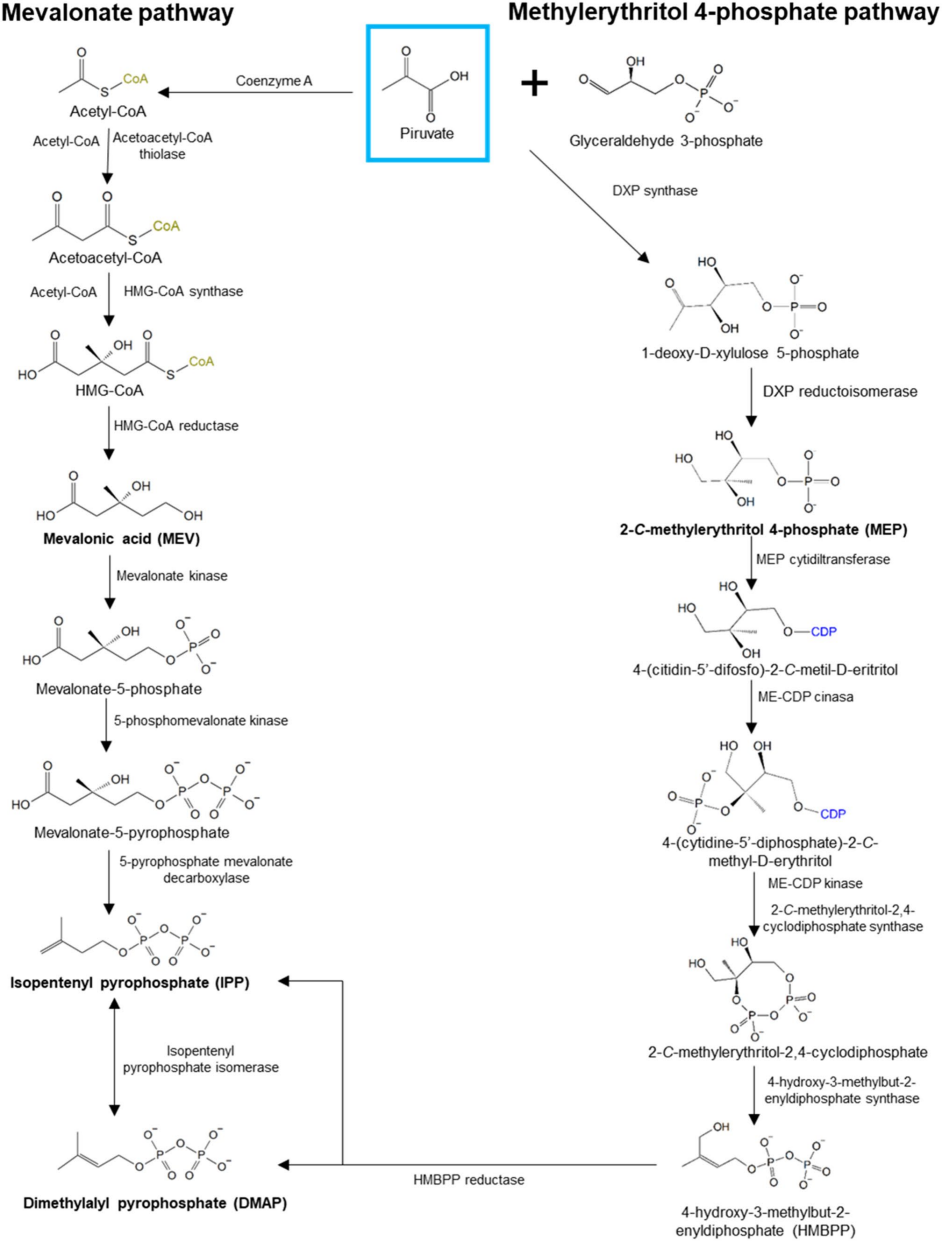
Synthesis routes of IPP and DMPP. Mevalonate pathway (left); 2-*C*-methylerythriol-4-phosphate pathway (right) are present in plants, algae, and bacteria. Modified from Kuzuyama and Seto 2012

Fungi generate IPP and DMAPP only through the mevalonate (MEV) pathway, whereas plants and bacteria also rely on the 2-*C*-methyl-D-erythritol 4-phosphate (MEP) pathway to produce them (Banerjee & Sharkey 2013; Quin et al. 2014; Rodríguez-Concepción & Boronat 2002; Schmidt-Dannert 2015; Schrader & Bohlmann 2015) (Fig. 1).

**Fig. 2:**
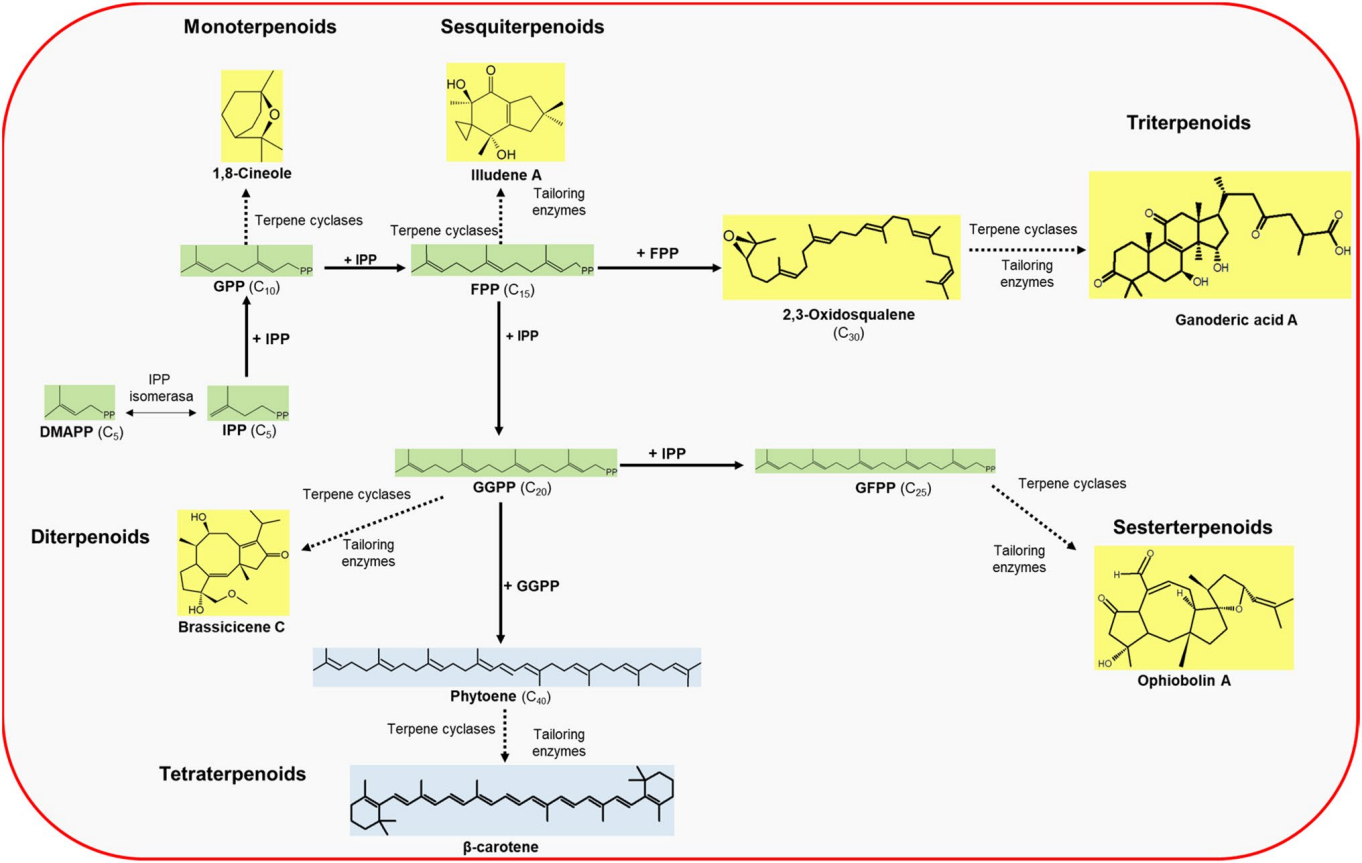
Biosynthesis of terpenoid compounds in fungi. Diphosphate isoprenoid precursors (green) are caused by the condensation of isopentenyl diphosphate (IPP) units. Later, terpene synthases (cyclases and tailoring enzymes) use isoprenoid precursors as substrates for the synthesis of terpenes (blue) and terpenoids (yellow), respectively

The mevalonate pathway starts with acetyl-CoA, coming from glycolysis or β-oxidation of fatty acids and obtained by the decarboxylation of pyruvate (Fig. 1) (Kuzuyama & Seto 2012; Nelson et al. 2005; Quin et al. 2014; Schmidt-Dannert 2015; Schrader & Bohlmann 2015). Once IPP is synthesized, it is used to obtain isoprenoid precursors: geranyl diphosphate (GPP), farnesyl diphosphate (FPP), and geranylgeranyl diphosphate (GGPP), among others (Fig. 2). These compounds are the substrates of the various terpene synthases involved in terpenoid biosynthesis (Nelson et al. 2005; Schmidt-Dannert 2015; Schrader & Bohlmann 2015).

## Terpene synthases

**Fig. 3:**
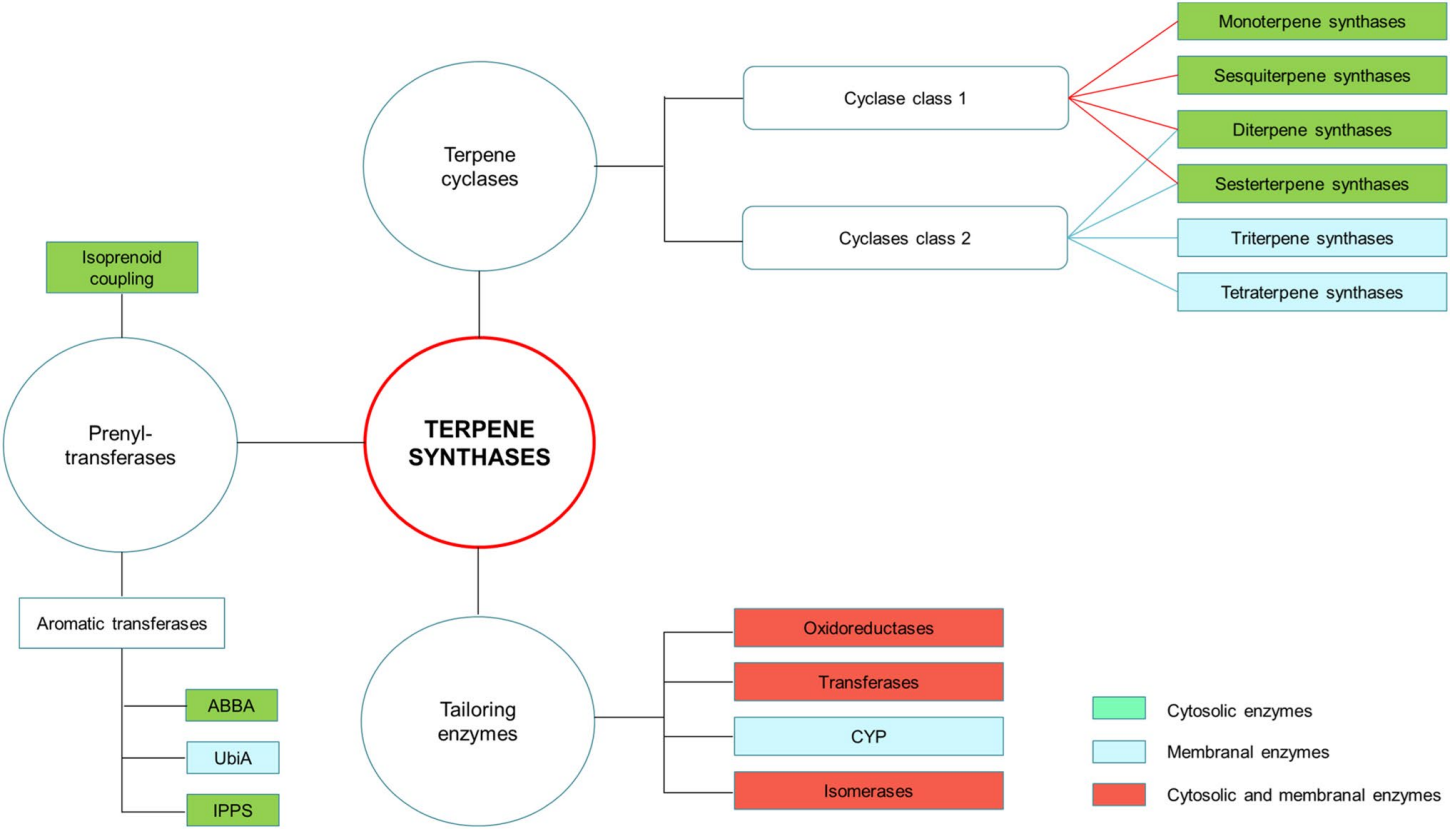
Classification of fungal terpene synthases. There are three main groups of terpene synthases: prenyltransferases (left), terpene cyclases (top), and tailoring enzymes (bottom). The colors in the enzyme subgroups indicate whether they are cytosolic (green) or membrane integral (blue). Enzymes that cannot be strictly classified into one or the other group are shown in red

The synthesis of terpenes occurs in diverse and complex steps, which include condensation, formation and stabilization of carbocations, and the addition of functional groups. The enzymes involved in synthesizing terpenes/oids are called terpene synthases, and they can be classified according to their function as prenyltransferases, terpene cyclases, and tailoring enzymes. The first step in the synthesis occurs after the condensation of the IPP and DMAPP units by isoprenoid coupling enzymes, giving rise to the precursors for terpenoids. These compounds are substrates for terpene synthases, such as prenyltransferases and terpene cyclases. Terpenecyclases are the ones that provide structure to the scaffold of the molecule. They can be classified according to the number of isoprene units contained in the isoprenoid precursor, which its substrate contains. Finally, tailoring enzymes act, which modify the structure generated by terpene cyclases by adding functional groups giving rise to terpenoids (Fig. 3) (Christianson 2008, 2017; Schrader & Bohlmann 2015; Wawrzyn et al. 2012).

### Prenyltransferases

Prenyltransferases are involved in the synthesis of direct terpenoid precursors, referred to in this review as isoprenoid precursors and terpenoids of mixed biosynthetic origin. All prenyltransferases have conserved aspartate-rich motifs that allow the binding of divalent metal ions involved in the elimination of diphosphate groups (Christianson 2008, 2017; Tello et al. 2008). The prenyltransferases are classified into two groups based on their function: the isoprenoid-coupled enzymes and the transferases that bind isoprenoid chains to molecules originating from other metabolic pathways (indoles, polyketides, among others) (Tagami et al. 2013; Tello et al. 2008).

## Isoprenoid coupling enzymes

These enzymes are involved in the elongation of the isoprenoid chain through the condensation of IPP/DMAPP units, leading to the formation of GPP, FPP, GGPP, GFPP, squalene, and phytoene. These enzymes have two conserved divalent metal ion binding motifs in the entrance of the active site cavity, which are rich in aspartate; the most common type of this motif is DDXXD (D: aspartate, X: any amino acid); in the process, the binding of 3 metal ions is required. Together with this, the presence of hydrogen bonds and another conserved motif with three arginine/lysine residues allow the removal of diphosphate from IPP/DMAPP to generate a carbocation and elongate the isoprenoid chain (Schmidt-Dannert 2015; Tagami et al. 2013; Tello et al. 2008).

## Aromatic Prenyltransferases

**Fig. 4:**
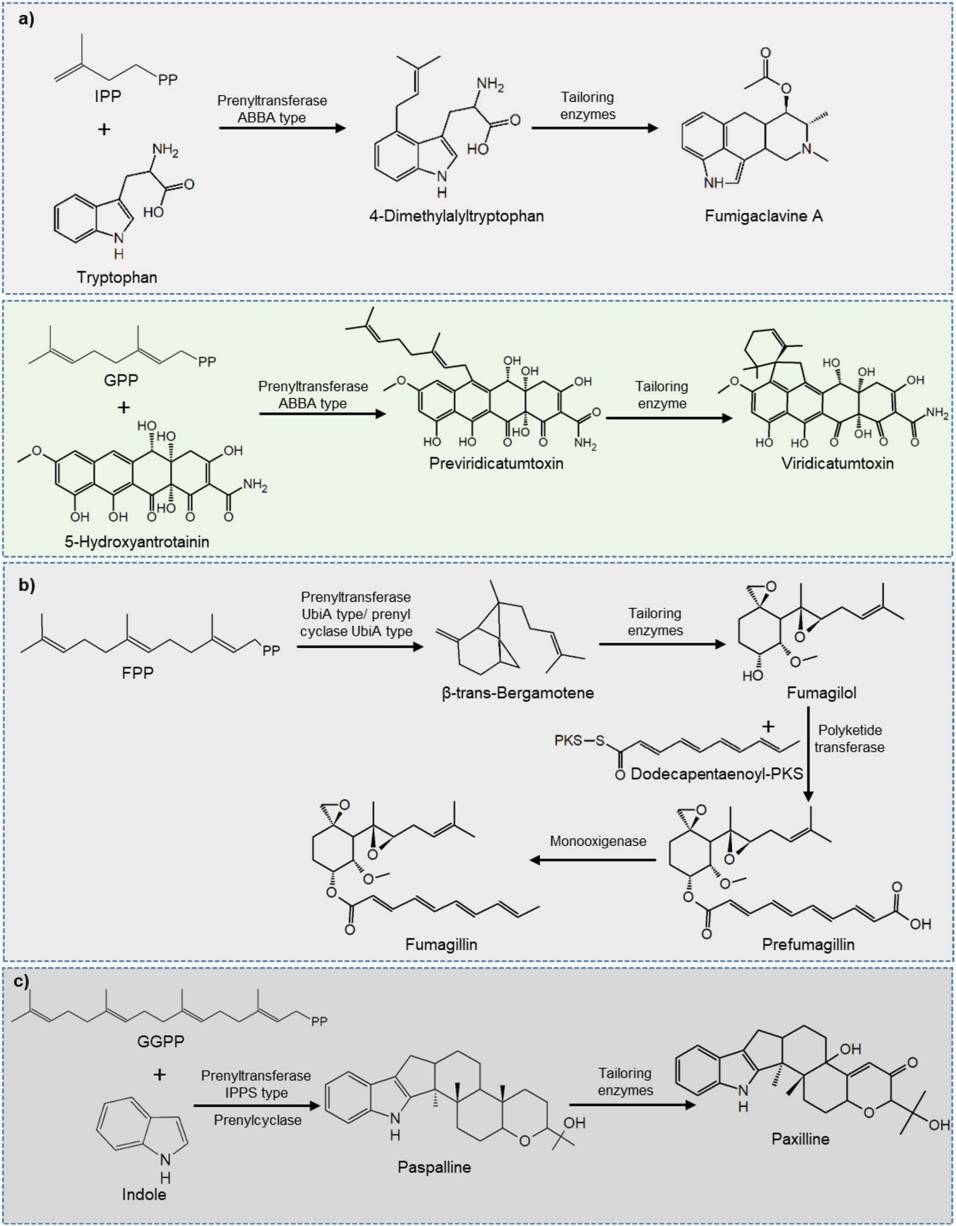
Classification of prenyltransferases. ABBA (**a**) prenyltransferases transfer isoprenoid chains to indole and polyketide structures. UbiA (**b**) can transfer cyclized FPP to other molecules such as fatty acids. IPPS types (**c**) transfer 20-carbon chains and require coupling with prenylcylases for their function. Modified from Schmidt-Dannert 2015

These enzymes are classified into three families (Fig. 4). The first is the ABBA-type enzymes; these are so named because they have a β-barrel structure consisting of repeated αββα elements (Schmidt-Dannert 2015; Christianson 2017; Tello et al. 2008). The prenyltransferases of this group transfer DMAPP and GPP to cyclic indoles or polyketides by generating a cation at C_1_ or in C_3_. Subsequently, the double bonds or electron-donating atoms (oxygen, nitrogen) of the receptor molecule attack the positive charge, condensing both structures (Fig. 4a) (Schmidt-Dannert 2015; Christianson 2017; Tagami et al. 2013; Tello et al. 2008).

The second family of prenyltransferases is the UbiA type, so named because they have a domain like the prenyltransferase involved in the synthesis of ubiquinone (coenzyme Q10), which transfer FPP to polyketide derivatives, generating meroterpenoids. These enzymes are membrane integral and can perform cyclization with the C_1_ of the isoprenoid chain (Fig. 4b) (Schmidt-Dannert 2015; Christianson 2017). Generally, the compounds formed have pharmacological activity, among which terretonins (Fukuda et al. 2014) and mycophenolic acid derivatives stand out (Golshayan et al. 2009).

The last family is the IPPS (isoprenyl diphosphate synthases) type. They transfer GGPP to indole structures, they have the ability to cycle the structure from C_1_, they also have a conserved amino acid motif D(D/E)XX(D/E), and they are cytosolic (Christianson 2017). All the compounds generated by these enzymes are mycotoxins, which have been obtained exclusively in ascomycetes and commonly require the complementary activity of other cyclases to give the final structure to the compound, for example, paxilline (Fig. 4c) (Schmidt-Dannert 2015; Christianson 2017).

## Terpene cyclase

**Fig. 5:**
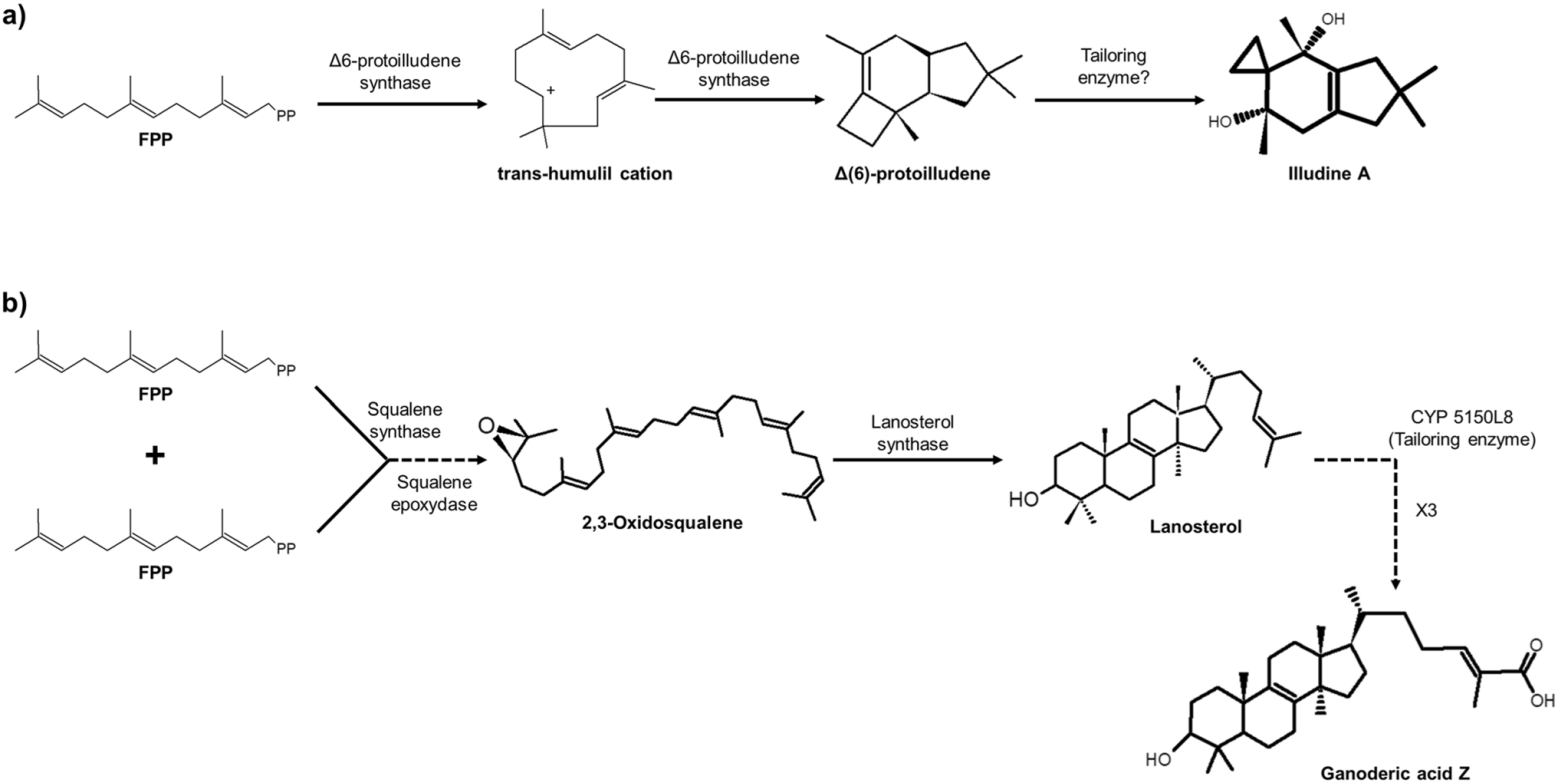
General synthesis of terpenoids mediated by type I (**a**) and type II (**b**) cyclases. Type I cyclases possess conserved sites [D(D/E)XX(D/E) and NSE/DTE] and are the only ones that can remove the diphosphate group and form a carbocation in the process of cycling the structure. Type II cyclases generate the carbocation to cyclize by activating the epoxide by donating a proton from a catalytic site aspartic acid (DCTAE). After obtaining the structure of the scaffold terpene, modifications by tailoring enzymes arise the terpenoids

**Fig. 6:**
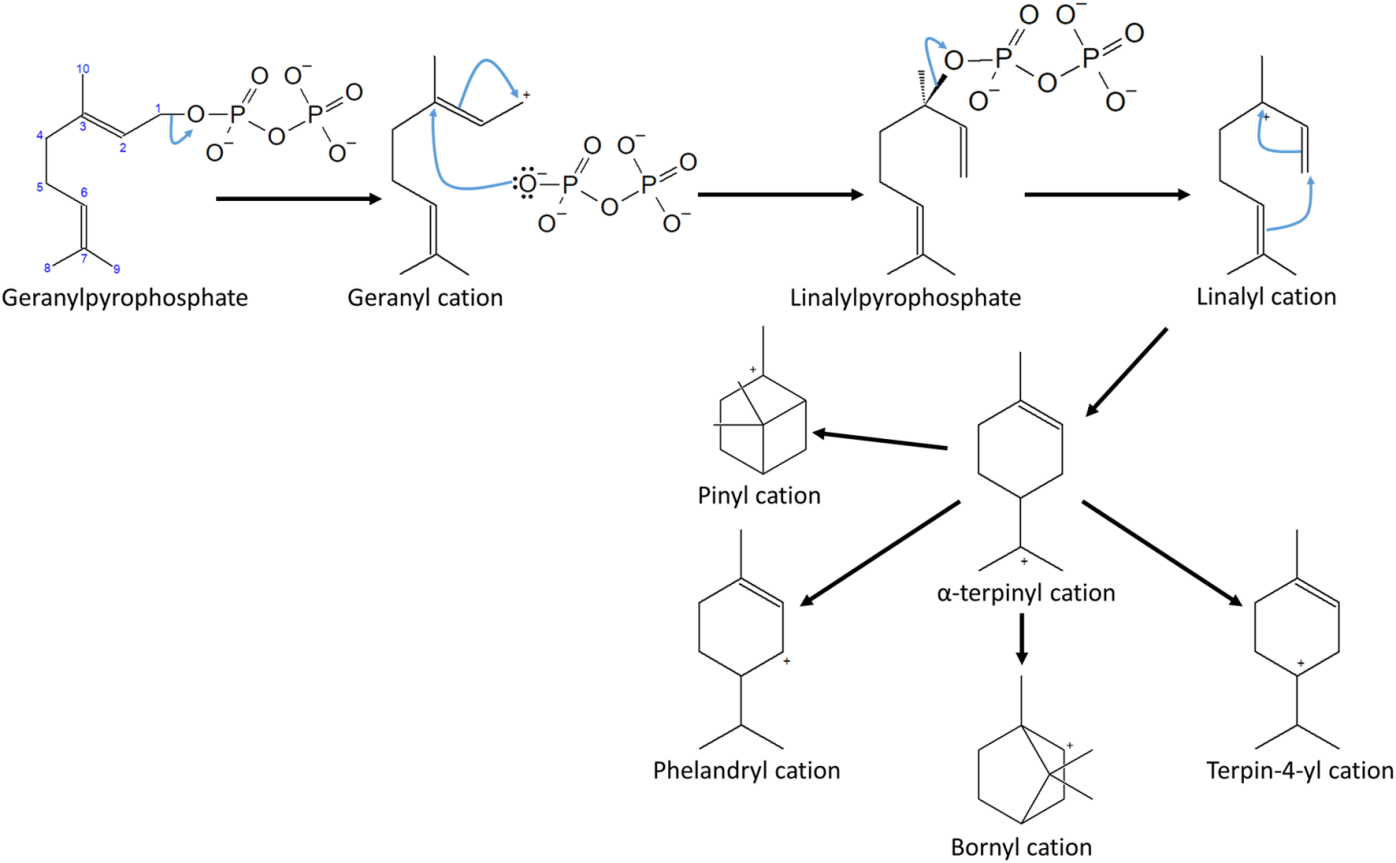
Monoterpene synthase cyclization mechanism. The formation of the linalyl cation gives rise to the formation of the α-terpinyl cation, the precursor of fungal cyclic monoterpenoids

Terpene cyclases (TC) are membrane enzymes that perform the function of cyclizing isoprenoid chains and are classified based on their reaction mechanism into two classes (Fig. 5). Class I TC, closely related to prenyltransferases, are characterized by an alpha-helix structure that forms the active site cavity and has two conserved regions of aspartate rich motif [D(D/E)XX(D/E)] and an NSE/DTE type. These cyclases carry out their activity by the formation of a secondary or tertiary carbocation, mediated by the cation of a divalent metal (generally Mg^2+^), which facilitates the removal of the diphosphate group, by a mechanism similar to that of prenyltransferases. Subsequently, a double bond (C=C) from another region in the chain attacks the carbocation by an electrophilic addition mechanism, generating the cyclized structure (Fig. 6). It is worth mentioning that only class I cyclases can remove the diphosphate group (Fig. 5a) (Christianson 2008, 2017; Quin et al. 2014; Schmidt-Dannert 2015).

Class II TCs present a double-barrel alpha-helix structure with a hydrophobic active site located in the middle and are subdivided into two subtypes: those that generate activation on the double bond, which present a conserved motif amino acid DXDD (bacteria and plants), and those that activate epoxide (fungi and animals) contain a catalytic aspartate in a DCTAE motif. The cyclization mechanism begins with adding a proton from aspartate to a double bond or epoxide, destabilizing the bonds and generating a carbocation that can be attacked by a double bond, allowing the structure to cycle. Fungi can synthesize terpenoids ranging from 10 to 40 carbon atoms; however, the most frequent are sesquiterpenes, diterpenes, and triterpenes (Fig. 5b) (Christianson 2017; Quin et al. 2014; Schmidt-Dannert 2015). Therefore, the studies have focused on their respective terpene synthases, which will be mentioned below.

### Monoterpene synthases

Although fungi mainly produce terpenes and terpenoids of 15 to 30 carbons, several genera have been identified, such as *Agarocybe, Tricholoma*, *Muscodor, Hypoxylon, Daldinia, Nemania, Genicolusporium*, capable of producing monoterpenes (Citron et al. 2012; Lauterbach et al. 2019; Macías-Rubalcava et al. 2010; Rapior et al. 1998; Rinkel et al. 2016; Shaw et al. 2015). To date, monoterpene synthases continue to be identified in fungi; for example in eight isolates of *Hypoxylon* sp. (brown rot fungus), the production of 1,8-cineole (eucalyptol) was detected, which had only been detected in plants, as well as the monoterpene synthases related to the synthesis (Shaw et al. 2015).

Monoterpene synthases belong to class I cyclases; that is, their cyclization mechanism depends on the elimination of the diphosphate group, giving rise to the formation of the carbocation in C_1_ (linalyl cation) and the subsequent cyclization of the C_1_–C_6_ structure. Once the structure is cyclized, the α-terpinyl cation is generated, a six-membered ring is generated, and the positive charge on the tertiary carbon (C7) of the isopropyl group is formed. From the α-terpinyl cation, the rest of the carbocations that make up the scaffolds of monoterpenoids are derived (Fig. 6) (Degenhardt et al. 2009; Rinkel et al. 2016).

### Sesquiterpene synthases

These enzymes use FPP as a substrate, and although all sesquiterpene cyclases recognize it, the mechanism and the resulting structure are particular for each case. Fungal sesquiterpene cyclases mainly use (*2E*,*6E*)-farnesyl diphosphate and molecules with different base structures have been found. First, there are the enzymes that use FPP and cycle it C_1_–C_10_ (germacrene A synthases, α-murolene synthases, and aristoloquene synthases) and those that cycle it C_1_–C_11_ (protoiludene synthases, presylphperfolan-8β-ol synthases, and koraiol synthases). FPP can be isomerized by enzymatic action by translocating the diphosphate group from C_1_ to C_3_ and the double bond from C_2_–C_3_ to C_1_–C_2_, forming (*3R*)-nerolidyl diphosphate (NPP).

**Fig. 7:**
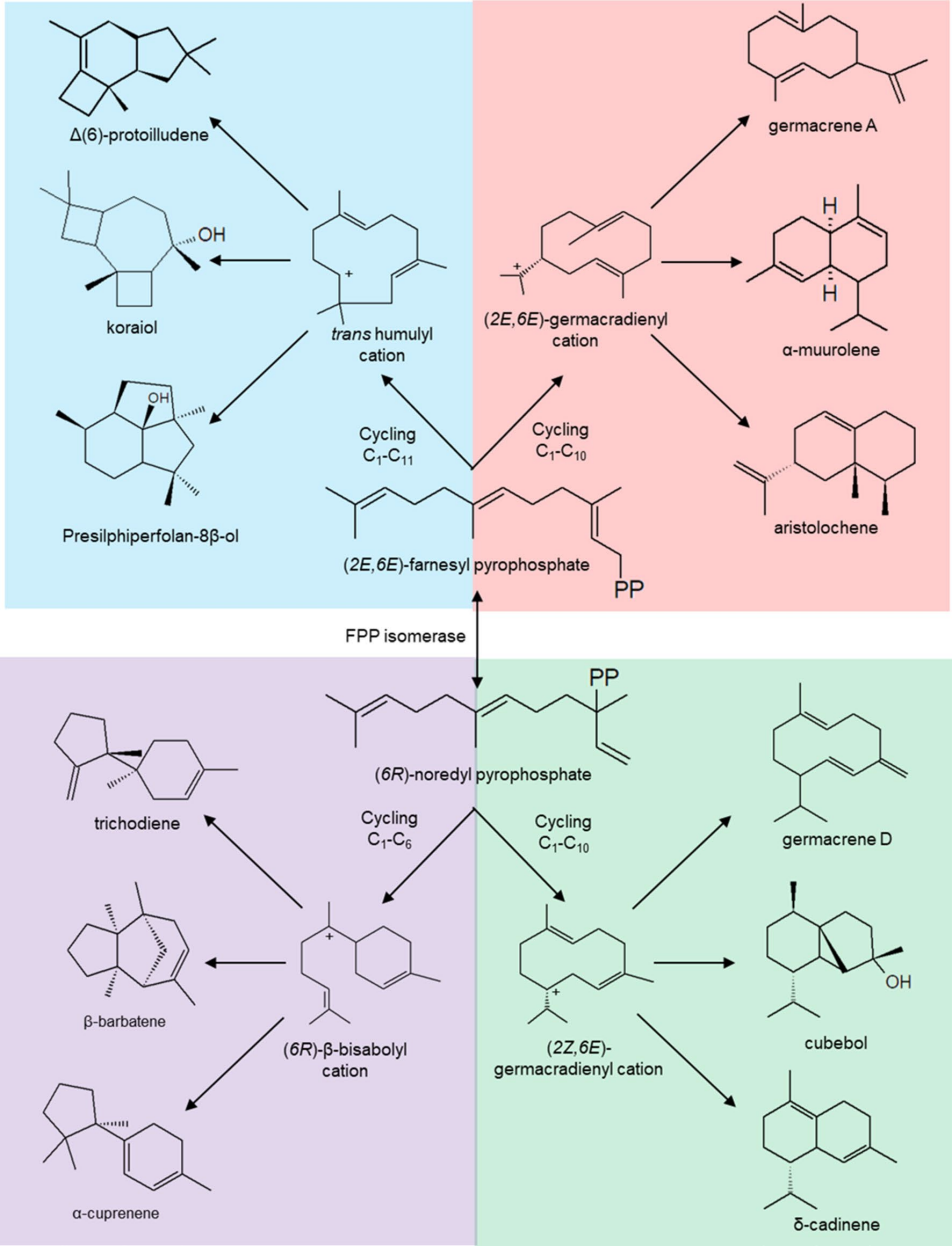
Types of cyclization and formation of the most common intermediate carbocations in synthesizing fungal sesquiterpenes. For the synthesis of these compounds, the most common substrate is (*2E, 6E*)-FPP (top), and only fungi that possess FPP isomerase can synthesize the derivatives of (*6R*)-noredyldiphosphate (bottom). Modified from Schmidt-Dannert 2015

NPP serves as a substrate for barbatene synthases, trichodyne synthases, longiborneol synthases, α-acorenol synthases, and α-cuprenene that cyclize C_1_–C_6_. NPP can also be cyclized into C_1_–C_10_, a mechanism employed by germacrene D synthases, epi-zonarene synthases, and cadinene synthases (Fig. 7) (Agger et al. 2009; Christianson 2017; Degenhardt et al. 2009; Quin et al. 2014; Schmidt-Dannert 2015). Some sesquiterpene synthases are promiscuous by substrates, such as germacrene A synthase (Cop1) from *Coprinus cinereus*, which recognizes FPP and NPP to produce germacrene A and germacrene D, respectively. It should be noted that although it is possible to classify these enzymes by the initial cyclization mechanism of the FPP or NPP structure, as the case may be, the carbocation stabilization mechanism is the crucial step in generating the different terpenic products (López-Gallego et al. 2010).

Other sesquiterpene synthases generate more than one product from a precursor, for example, the α-murolene synthases from *Omphalotus olearius*, which can mainly synthesize α-murolene and germacrene A in a lower proportion (Wawrzyn et al. 2012). In addition, there are reports of sesquiterpene synthases in vitro, which can recognize GPP as a substrate, forming monoterpenoids in the absence of FPP (López-Gallego et al. 2010). However, this has not been demonstrated in vivo; for this reason, further studies are required to understand the conditions that allow sesquiterpene synthases to synthesize monoterpenes.

### Diterpene synthases & sesterpene synthases

**Fig. 8:**
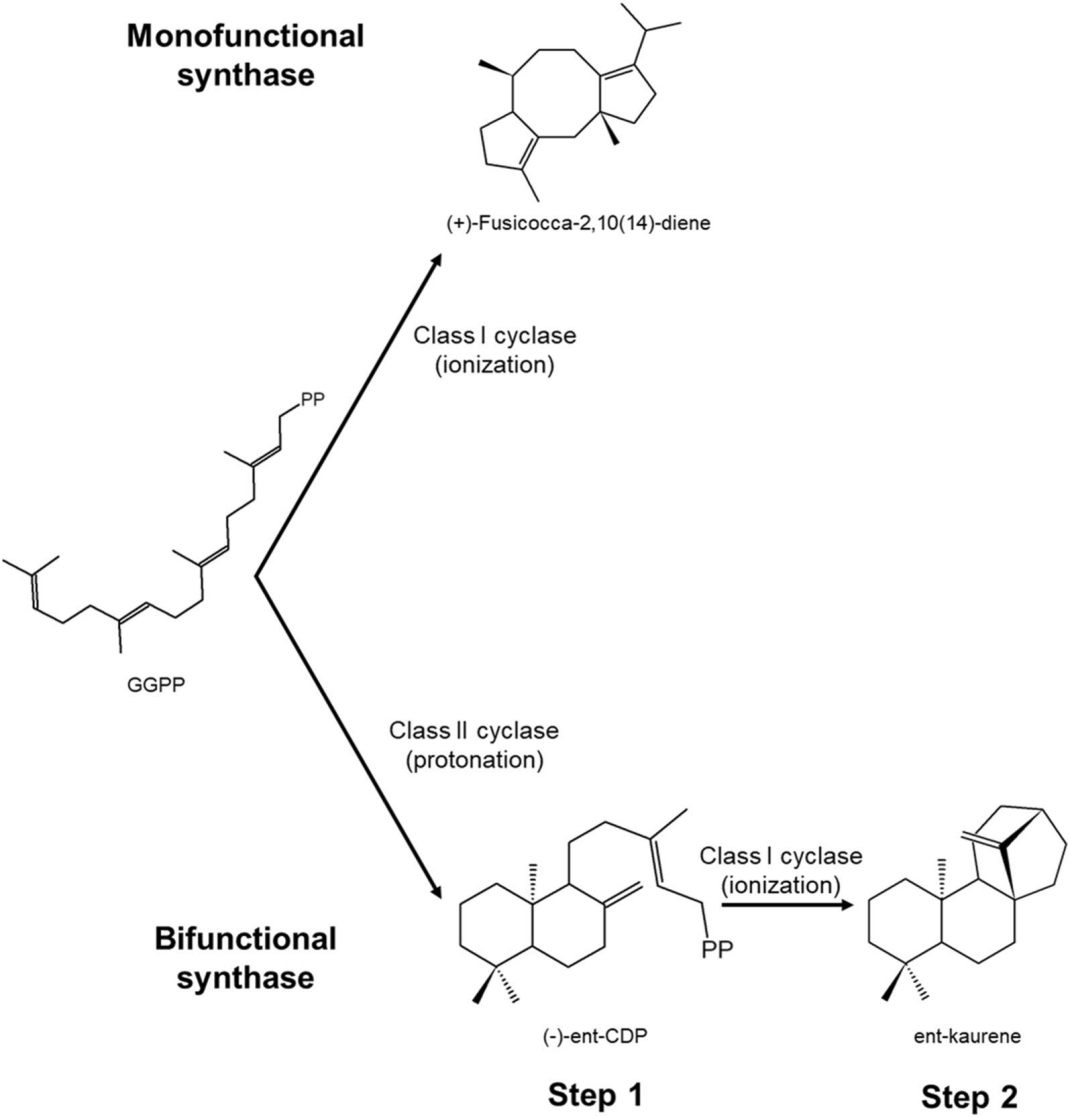
Types of fungal diterpene and sesterpene synthases. Monofunctional diterpene/sesterpene synthases have type I cyclase activity. While the bifunctional ones have two catalytic sites, one characteristic of type II cyclase and the other of type I cyclase. Modified from Quin et al. 2014

Diterpenoids and sesterpenoids are obtained from GGPP and GFPP, respectively. The process can be carried out in two ways; by modifying GGPP/GFPP by the activity of a class I terpene cyclase (monofunctional) or by unique enzymes in fungi called chimeric or bifunctional-terpene synthases (Fig. 8) (Christianson 2017; Degenhardt et al. 2009; Mitsuhashi & Abe 2018; Peters 2010; Quin et al. 2014; Schmidt-Dannert 2015). An example of the first case is the synthesis of ( +)-fusicocca-2,10(14)-diene by ( +)-fusicocca-2,10(14)-diene synthase, a diterpene cyclase of class I, which generates GGPP cycling between C_1_ and C_11_ (Fig. 8a). Some diterpene synthases are also promiscuous to recognize substrates, particularly monofunctional ones, which are capable of recognizing the 25-carbon isoprenoid chain, geranylfarnesyl diphosphate (GFPP), and cycling the structure, similar to what happened with fusicocca-2,10(14)-diene synthases, to form ophiobolins (sesterpenoids) (Quin et al. 2014; Schmidt-Dannert 2015). As with sesquiterpene synthases, these enzymes have conserved cyclization mechanisms. Still, the ability of synthase to stabilize carbocation is responsible for forming specific compounds.

**Fig. 9:**
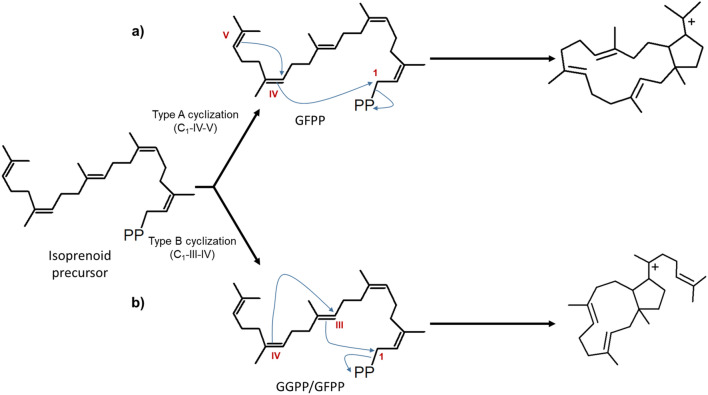
Types of cyclization of prenyltransferases-terpene synthases (PTTS). The type A (**a**) cyclization mechanism occurs only in synthesizing sesterpenoids. Type B (**b**) cyclization can occur for synthesizing diterpenoids or sesterpenoids

On the other hand, chimeric terpene cyclase class I-class II enzymes, as their name indicates, have a catalytic site of class I cyclase and another of class II cyclase; these carry out the synthesis of all the diterpenes of the type labdane and kaurene (Peters 2010). Regarding the reaction mechanism, it is expected that in the first place, deprotonation occurs on a double bond to generate the first cyclization (cyclase class II), immediately the catalytic site of cyclase class I comes into action to subtract the diphosphate and ends the molecule cyclization. Bifunctional diterpene synthases have two catalytic sites that carry out two different reactions on isoprenoid structures. For example, the synthesis of ent-kaurene (precursor of gibberellins) by ent-kaurene synthase (Fig. 8b) (Peters 2010; Quin et al. 2014; Schmidt-Dannert 2015). To date, two main groups of chimeric terpene synthases are known: prenyltransferases-terpene synthases (PTTS) and class I-class II terpene cyclases. Within the first group are the prenyltransferases- class I terpene cyclases and prenyltransferases- class II terpene cyclases, which have a catalytic domain towards the C-terminal region corresponding to prenyltransferase activity and towards the N-terminal region have a domain with terpene cyclase activity (Chen et al. 2016; Mitsuhashi & Abe 2018). In turn, class I prenyltransferases-terpene cyclases are classified into two clades based on their initial cyclization mechanism, type A and type B (Mitsuhashi & Abe 2018). Type A cyclization, also called C_1_-IV-V, has only been observed in PTTS involved in synthesizing sesterpenoids due to the necessary length in the isoprenoid chain. The process begins with the generation of the initial carbocation in C_1_ and later, the enzyme orients the double bond present in C_14_–C_15_ (double bond IV), transferring the carbocation to C_14_, which in turn is attacked by the C_18_–C_19_ double bond (double bond V) (Fig. 9a). Cyclization type B, or C_1_-III-IV, can occur both in the GGPP chain and in GFPP; Once the formation of the carbocation in C_1_ has occurred, it is attacked by the C_10_–C_11_ double bond, and the first cyclization step ends by the attack of the C_14_–C_15_ double bond to the new carbocation generated (Fig. 9b).

### Triterpene synthases

Triterpene synthases are class II cyclases and have a high substrate specificity. There are important differences between the triterpene synthases of eukaryotes and prokaryotes since in the latter, the substrate is squalene, while in those of eukaryotes, the substrate is 2,3-oxidoesqualene, generated by another enzyme called squalene monooxygenase (Dupont et al. 2012; Mitsuguchi et al. 2009; Quin et al. 2014; Schmidt-Dannert 2015; Tholl 2015); for this reason, eukaryotic triterpene synthases are also called squalene oxide cyclases. The synthesis of triterpenoids is of utmost importance in eukaryotes since sterols that are part of the cell membrane are synthesized from them, and deficiencies in the synthesis of these compounds can limit the tolerance of the organism to stress and cell proliferation (Jordá & Puig 2020; Szkopińska et al. 1993).

Plant triterpene synthases are the most varied, which in these organisms gives rise to a great diversity of triterpenoids with different base structures (Tholl 2015). On the other hand, fungi have few oxidosqualene cyclases, among which lanosterol synthase stands out, involved in ergosterol synthesis; therefore, the diversification of triterpenoids is mainly due to the tailoring enzymes they possess (Mitsuguchi et al. 2009).

**Fig. 10:**
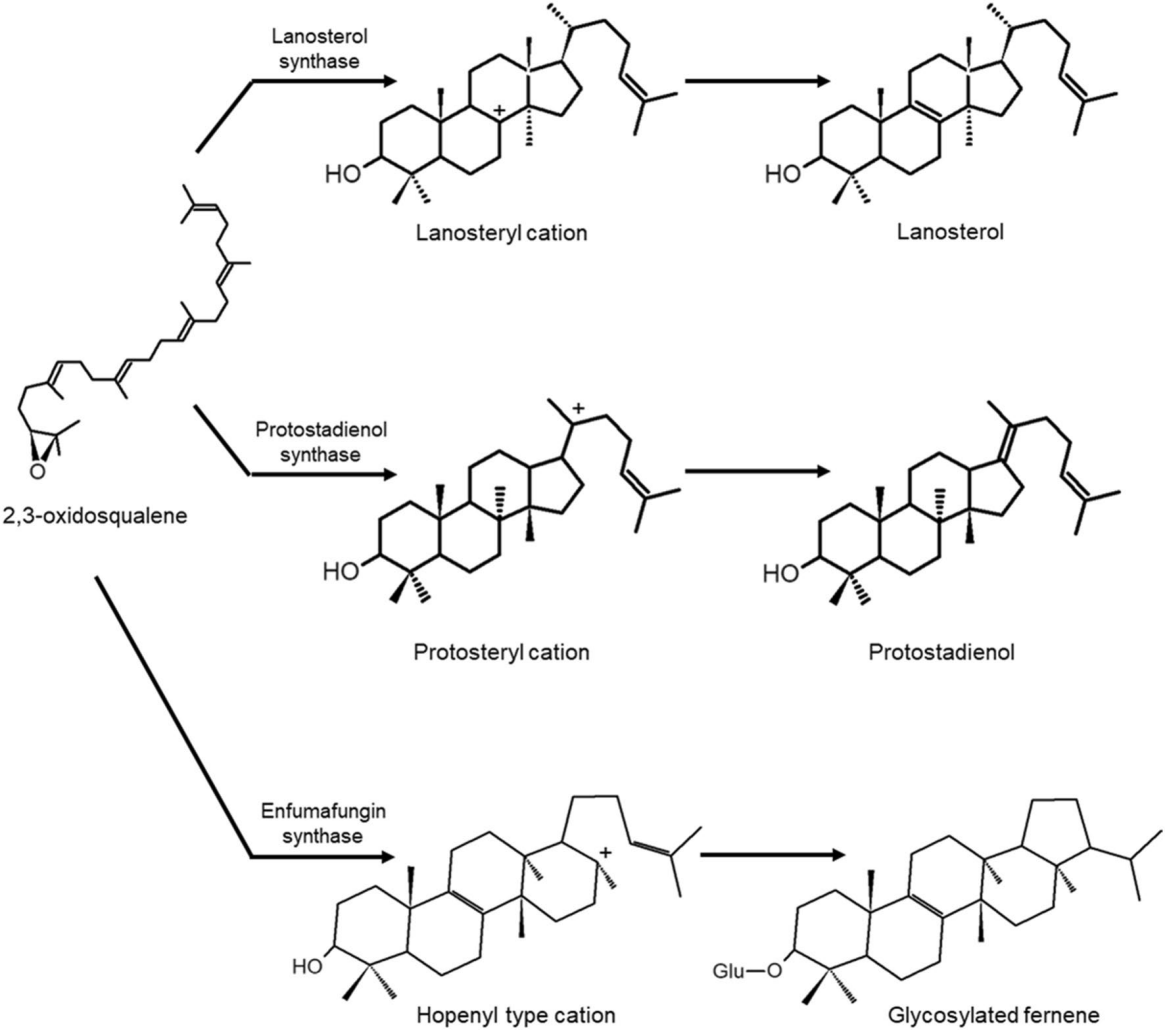
Known triterpene synthesis intermediates in fungi

Most of the triterpenoids of pharmacological interest produced by basidiomycetes originate from lanosterol (Kimura et al. 2009), while in filamentous fungi, most originate from protostadienol (Fig. 10). It has been proposed that the first carbocation generated upon cyclizing the 2,3-oxidoesqualene structure is that of the protosteryl, but lanosterol synthase facilitates the translocation of the positive charge to C_8_ to form the lanosteryl cation (Kimura et al. 2009; Schmidt-Dannert 2015). In addition, a new type of squalene oxide cyclase was discovered, enfamufungin synthase, which has bifunctional activity with one catalytic site for cyclization and another with transferase activity, forming glucosidated fernene (Kimura et al. 2009). The mechanism by which this cyclase forms a 5-ring structure is unknown. Still, it has been proposed that due to its similarity with hopene, the formation mechanism must be very similar (Fig. 10) (Kuhnert et al. 2018).

### Tetraterpene synthases

The synthesis of tetraterpenes in fungi is related to obtaining carotenoids, which have protective functions against oxidative stress and UV radiation and provide pigmentation. These compounds originate from phytoene, which is obtained by the condensation of two GGPP units and is carried out by phytoene synthase. Carotenoids can be linear or cyclic tetraterpenoids; in the first case, phytoene is a substrate for tailoring enzymes that will modify the structure's double bonds without generating cycles. While for cyclic compounds, type II cyclases use phytoene as a substrate and carry out their activity through mechanisms like triterpene cyclases (Avalos and Carmen Limón 2015; Lin & Xu 2020).

It is evident that there are very particular cyclization mechanisms for each type of terpene synthase, which is an important part of generating various terpenoid structures. A particularity of fungal prenyltransferases and terpene synthases is that they are precise for the synthesis of terpenoids (Kuhnert et al. 2018); however, there are cases of bifunctional enzymes that can recognize more than one substrate or generate more than one product from a substrate (Quin et al. 2014; Schmidt-Dannert 2015).

## Tailoring enzymes

This group comprises a wide variety of enzymes with different catalytic activities: oxidoreductases, cytochromes P450 (CYP), transferases, and isomerases, whose substrate is the product generated by terpene cyclases (Quin et al. 2014; Schmidt-Dannert 2015). For example, in *Omphalotus olearius*, 21 sesquiterpenoids have been identified, of which 20 correspond to derivatives of Δ6-protoilludene, also showing that all the structures underwent at least one oxidation process (Christianson 2017; Wawrzyn et al. 2012).

Although CYPs are the main tailoring enzymes, since they are the most abundant and are involved in modifications to generate terpenoids, other modifications such as esterification, glycosylation, alkoxylation, among others, are frequent in this type of compounds. Transferases are the enzymes involved in forming esters (acyltransferases) and glycosides (glycosyltransferases). In the case where terpenoid structures are condensed with alkaloids or polyketides, the transfer process of the isoprenoid component is mediated by prenyltransferases, which were previously discussed (Christianson 2017; Quin et al. 2014; Schmidt-Dannert 2015; Tagami et al. 2013; Tello et al. 2008).

Regarding their intracellular location, except CYPs wich are exclusively membrane and found in the endoplasmic reticulum, tailoring enzymes can be cytosolic, and membrane-attached. In the synthesis of terpenoids, modifications occur in more than one step and can be carried out by one or more tailoring enzymes sequentially. Therefore, it is common for terpenoids to be related by the same synthetic route, as they are intermediaries in the different modification steps by tailoring enzymes (Mitsuguchi et al. 2009; Quin et al. 2014; Schmidt-Dannert 2015).

## Prediction of terpenoid synthesis from the fungal genome

The most abundant secondary metabolites in fungi are terpenoids, of which sesqui and triterpenoids are the main ones (Baby et al. 2015; Caruthers et al. 2000; Lim et al. 2012; Martin et al. 2003; Quin et al. 2014; Schmidt-Dannert 2015; Wang et al. 2016). Currently, the analysis of the complete genomes of fungi is used as a tool for the prediction of the metabolic profile of organisms, particularly terpenoides (Christianson 2017; Lim et al. 2012; Wang et al. 2016). In these genomic and transcriptomic analyzes, it has been observed that many of the genes involved in the synthesis of secondary metabolites, particularly the terpenoids, are grouped in co-located blocks (clusters); which has made it easier to correlate the enzymes involved in the synthesis pathways with their expression (Christianson 2017; Lim et al. 2012; Wang et al. 2016). However, terpene synthases (cyclases and tailoring enzymes) have been found, which are not found in clusters, but co-express with prenyltransferases that elongate the isoprenoid chain (Lim et al. 2012; Wang et al. 2016; Wawrzyn et al. 2012).

This information represents a significant advance in the knowledge of fungal terpenoid synthesis since from these data it has been possible to predict the type of terpene synthases in fungi. In addition, the knowledge of the organization of genes and their co-expression has allowed us to better understand the processes of regulation of the transcription of the different enzymes involved in the synthesis of terpenoids. With this knowledge, strategies can be implemented to facilitate the selection of the organism and the cultivation conditions necessary to direct the biosynthesis of specific terpenoids.

## Regulation of terpenoid metabolism

The regulation in the production of terpenoids has been studied mainly in plants, where it has been observed that some factors, such as pH, humidity, and salinity of the soil, affect the synthesis of these molecules (Martin et al. 2003). However, in fungi, the response in terms of terpenoid production, depending on external stimuli, differs in some of the control points with respect to plants, which will be discussed below.

## Inhibition of mevalonate synthesis

**Fig. 11:**
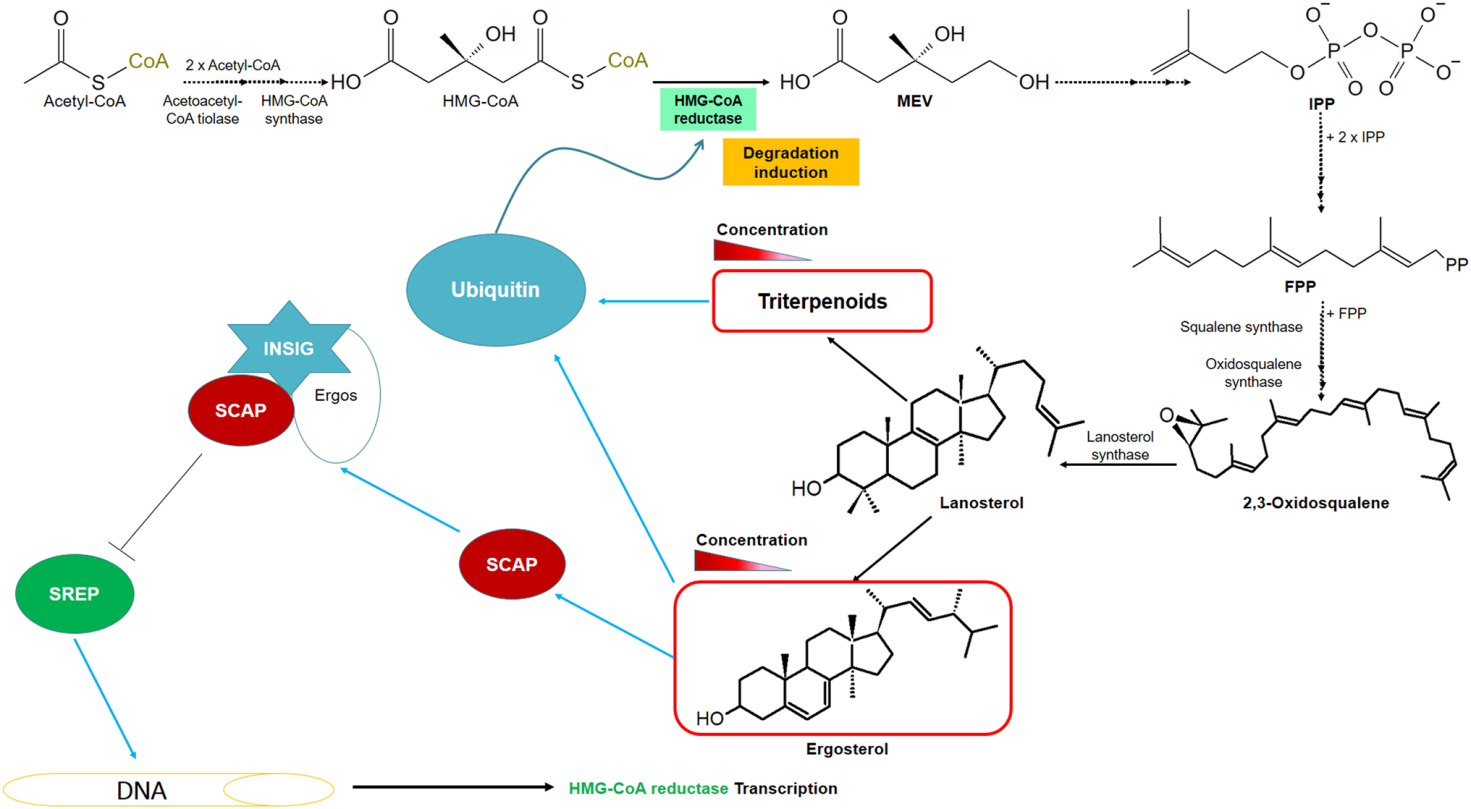
Regulation of the terpenoid synthesis pathway by degradation of HMG-CoA reductase

One of the main points of regulation in the synthesis of terpenoids is the inhibition of 3-hydroxy-3-methylglutaryl-CoA reductase (HMG-CoA reductase). The accumulation of some metabolites at the end of the synthesis pathway of terpenoids and sterols affects the regulation of HMG-CoA reductase (Dhingra & Cramer 2017; Johnson & DeBose-Boyd 2018; Wang et al. 2015). By inhibiting HMG-CoA reductase, the mevalonic acid pathway for the synthesis of IPP is blocked (Fig. 1) and terpenes. Both ergosterol and some terpenoids favor the ubiquitination of HMG-CoA reductase in the endoplasmic reticulum, leading to the enzyme's degradation (Fig. 11) (Johnson and DeBose-Boyd 2018). On the other hand, the concentration of ergosterol is detected by a protein complex consisting of a transcription factor called sterol regulatory element-binding protein (SREBP) and a protein that binds to SREBP, called SREBP cleavage-activating protein (SCAP). When there are low levels of ergosterol, SREBP translocates from the endoplasmic reticulum to the nucleus to bind to the regulatory elements of sterols located in the promoter region of HMG-CoA reductase. When the endoplasmic reticulum reaches a sufficient concentration of ergosterol, the latter binds to the SCAP protein, and this, in turn, attaches to the proteins of the insulin-induced genes (INSIG) of the endoplasmic reticulum; consequently, the SREBP factor cannot leave the lattice to carry out its function (Fig. 11) (Dhingra and Cramer 2017; Johnson and DeBose-Boyd 2018; Sun et al. 2005; Wang et al. 2015; Yang et al. 2002).

## Tissue-specific terpene synthases

Fungi, and specifically basidiomycetes, have different stages of development, where differentiated tissues are formed that present specific patterns of epigenetically regulated gene expression (Borgognone et al. 2018; Vonk and Ohm 2021). The differentiation of the tissues brings changes in the expression of terpene synthases and, consequently, in the terpenoids produced (Chen et al. 2012; Chen et al. 2017; Satrauss and Reyes-Dominguez 2011).

In a study by Chen et al. (2012), the transcriptome of *Ganoderma lucidum*, one of the most important fungi from traditional Asian medicine, was analyzed at three stages of development: mycelium, primordium, and fruiting body. It was shown that *G. lucidum* in the mycelium expressed 429 genes differentially, while in the primordium and the fruiting body, 57 and 91 genes were expressed differentially, respectively. However, when comparing the profile of terpenoids produced in each stage of development, it was observed that in the mycelium, the production is lower than in the primordium and fruiting body. Likewise, the expression level of CYPs was quantified in the three stages of development; in this case, the result showed that particular CYP groups are expressed in each case, the mycelium being the one with a lower amount of these expressed enzymes (Chen et al. 2012). This may be related to the amount of terpenoids detected in each development phase since as mentioned above, CYPs play a fundamental role in the diversification of terpenoids. In the case of *Hericum erinacius*, something striking is that it presents in the mycelium (both primary and secondary) the highest level of transcription of the genes involved in the synthesis of sesquiterpenoids compared to the fruiting body. While in the fruiting body, the overexpressed genes are involved in synthesizing diterpenoides (Chen et al. 2017).

In the organisms there are genes that are constitutively expressed genes, while others are particularly expressed in each tissue, which in this case can be associated with tissue-specific secondary metabolism, which has been reported commonly observed in plants (Dubey & Jeon 2017; Yan et al. 2022). Remembering that in the formation of different developmental structures in organisms, and particularly in fungi, tissue-specific gene regulation occurs. Epigenetic regulatory mechanisms include: DNA methylation and histone modification, chromatin remodeling, non-coding RNA silencing (Jaenisch & Bird 2003; Dubey & Jeon 2017). Based on the above, it is important to understand that although a basidiomycetes produces some terpenoids when found in nature (with all its developmental structures), they will not necessarily be produced under laboratory conditions, particularly when the fungus are submerged or solid cultured. This is extremely interesting since understanding how to manipulate the environmental signals that induce the differentiation of the structures of medicinal mushrooms can be a potential tool for the differential production of terpenoids of interest.

## Regulation of terpene synthase transcription

In nature, regulation of secondary metabolism gene transcription is a response commonly observed by organisms to different environmental (temperature, light, pH, reactive species) and nutritional (carbon, nitrogen, oxygen source) stimuli (Table 1) (Espeso & Peñalva 1996; Jiao et al. 2008; Kawakami et al. 2014; Kenne et al. 2018; Lind et al. 2016; Nazari et al. 2016; Pruss et al. 2014). As mentioned above, many of the genes involved in the secondary metabolite synthesis pathway form clusters co-regulated at the transcription level. In the case of terpenoid synthesis, it has been seen that some of these factors impact the transcription of genes involved in their biosynthesis. The effect of temperature on the synthesis of trichothecenes T2 and HT-2 was evaluated in two species of *Fusarium* (*Fusarium sporotrhichioides* and *Fusarium langsethiae*) (Nazari et al. 2016). This study revealed that the highest production of trichothecenes in both species is obtained at temperatures close to 15 °C, while above 30 °C, the synthesis of these compounds is reduced (Nazari et al. 2016). This response is mediated by the Velvet complex, composed of subunit A (VeA), subunit B (VelB), and the regulator of loss of expression of aflR A (LaeA); this complex had been initially described and related to the response to different light intensities. The VeA-VelB complex enters the nucleus after stimulation, and once there, VeA interacts with LaeA (a chromatin-remodeling protein), regulating the transcription of secondary metabolism genes (Lind et al. 2016).

**Table 1:** Factors involved in the regulation of terpenoid synthesis

Parameter	Regulatory mediator	Effect
State of development	Chromatin conformation	Selective expression of terpene synthases characteristic of the tissue type
Some terpenoid compounds and sterols	SRBP-SCAP protein complex	Suppression of HMG-CoA reductase transcription. Inhibits the synthesis of IPP and all its derivatives
Light and temperature	Velvet complex (VeA, VelB and LaeA)	Reshapes chromatin to facilitate gene transcription of some terpene synthases
Reactive oxygen species and pH	bZIP proteins	Induces transcription of terpene synthase genes that have the recognition sequence of bZIP proteins
Nitrates and low NH_3_ concentration	AreA	Induces transcription of terpene synthase genes that possess the AreA recognition sequence
Different carbohydrates	¿CreA/CRE1 AMPc?	The regulation mechanism is unknown with precision, but according to the proposals of the various authors, CreA or cAMP may be regulating the expression of the genes involved in the synthesis of some terpenoids
Divalent ion type (Mg^2+^, Ca^2+^, Cu^2+^)	Sesquiterpene synthase activity	In vitro*,* modifies the specificity of product formation by sesquiterpene synthases
NADP^+^/NADPH ratio	CYP monooxygenase and oxidoreductase activity	Decreases the activity of CYPs and oxidoreductases, preventing the oxidation of terpenes

Other studies have observed that the synthesis of aflatoxins is a mechanism to fight against reactive oxygen species (ROS) in filamentous fungi, such as *Aspergillus parasitucus* (Kenne et al. 2018). The synthesis mechanism of these compounds by the response to ROS and pH is mediated by the transcription factors bZIP (proteins with a leucine zipper domain, whose consensus sequence is: 5'-TGACGTCA-3 '), also involved in the transcription of other virulence and sexual development factors in filamentous fungi (Huang et al. 2015; Kenne et al. 2018); This is because, in their natural environment, pathogenic fungi and plant symbionts must contend against the ROS produced by these in the colonization process (Kenne et al. 2018)**.**

On the other hand, one of the most relevant nutrients in the production of fungal terpenoids is nitrogen. Various nitrogen sources have been shown to affect the synthesis of the terpenoid deoxynivalenol and gibberellins in *Fusarium* species, mediated by the AreA transcription factor (Hou et al. 2015; Kawakami et al. 2014; Mihlan et al. 2003). AreA accumulates in the nucleus when there are low ammonia levels and is induced by nitrate; among its functions, AreA increases the synthesis of permeases to capture nitrogen from the medium. In these studies, it was possible to show that when generating *ΔareA* mutants, there was a significant reduction of up to 90% in the synthesis of trichothecenes and gibberellins. When complementing the mutants with the AreA gene, the synthesis of terpenoids was re-established at the same levels as the native strain (Hou et al. 2015; Mihlan et al. 2003).

Another essential nutrient in the production of terpenoids is the carbon sources.It has been observed in various studies that in fungi, the carbon source used in the culture medium impacts the synthesis of various secondary metabolites (Cui et al. 2015; Diamantopoulou et al. 2014; Dilks et al. 2019; Gutiérrez et al. 1999; Hu et al. 2017; Jiao et al. 2008; Kawakami et al. 2014; Miyanga 2017; Portnoy et al. 2011).For example, the use of glucose in the culture of filamentous fungi has a negative effect on the production of certain secondary metabolites, such as penicillins (Gutiérrez et al. 1999), but with some polyketides, such as sorbicillinoids, it does not show a repressive effect (Zhang et al. 2023). This shows that the regulation by the carbon source on secondary metabolism is complex and does not apply equally to the different types of compounds.

In the case of terpenoids, it has been observed that the synthesis of trichothecene derivatives (deoxynivalenol) is induced by the use of sucrose and other fructooligosaccharides (1-kestose and nystose) in *Fusarium graminearum* and *Fusarium asiaticum*. In these studies, the use of glucose in combination with sucrose was evaluated and it was observed that deoxynivalenol continued to be produced, for which a repression effect was not appreciated when using glucose, but it seems to be an induction by the use of sucrose (Jiao et al. 2008; Kawakami et al. 2014).

Based on the identification of consensus sequences in the promoter regions of the genes of the enzymes of the biosynthetic pathway for the catabolic repressor CreA (CRE1), some authors have been suggested that the mechanism of regulation, by carbon source, of the synthesis of secondary metabolites, including terpenoids, is mediated by this protein (Dhingra & Cramer 2017; Johnson & DeBose-Boyd 2018; Kawakami et al. 2014). CreA/CRE1 is a global regulator whose primary known function is as an induced catabolic repressor, mainly by glucose, inhibiting the synthesis of enzymes and other proteins involved in the metabolism of complex carbon sources. The genes that CRE1 regulates have been studied in *Trichoderma reesei*, generating mutants of this fungus, deficient in the catabolic repressor. Among the secondary metabolism genes that increased their transcription are CYP monooxygenases and an ortholog of *paxU*, involved in synthesizing indole diterpenoids, such as paxilline (Portnoy et al. 2011). It is interesting to note that not all secondary metabolism genes are affected in the same way since, in this study, it was observed that another group of genes, particularly those related to the synthesis of polyketides, decrease their transcription in the absence of CRE1. Since CreA/CRE1 is a global regulator, it is possible that the effect observed in transcriptional changes is not associated with a direct regulation by CreA/CRE1 but rather that metabolic changes at different levels influence the synthesis of terpenoids. This taking into account that in the *Trichoderma reesei* ΔCRE1 mutant, a decrease in growth was observed compared to the native strain (Portnoy et al. 2011).

However, in the case of the synthesis of trichothecenes in the different *Fusarium* strains, it was observed that using only glucose did not produce these compounds. But when combined with sucrose, the production of trichothecenes occurs. This suggests, in the first instance, that there is no Cre1-mediated catabolic repression. And this was confirmed by analyzing at the transcriptional level, some genes of the trichothecene synthesis cluster, *TRI4* (cytochrome P450 monooxygenase), *TRI5* (trichodiene synthase), *TRI6* (pathway regulator), and *TRI10* (transcription factor) were analyzed while using glucose or sucrose as a carbon source in the culture. In this case, an effect was observed in transcription only in the *TRI4* and *TRI5* genes. When using sucrose, the transcription of these two genes was induced on the third day of culture, while when using glucose, no increase in transcription was observed in the entire cultivation time. Something that is interesting in this study is that the transcription levels of the *TRI6* and *TRI10* genes do not show differences when using glucose or sucrose as a carbon source, both genes, and particularly *TRI6*, are crucial for the synthesis of deoxynivalenol (Jiao et al. 2008; Kawakami et al. 2014).

Another hypothesis proposed regarding the regulation of the synthesis of deoxynivalenol is related to the response of the different levels of cAMP. In the study carried out by Jiang et al. (2016) it was observed that the presence of exogenous cAMP in the PH-1 strain of *Fusarium graminearum* and various mutants induces the synthesis of deoxynivalenol. In this study, mutants of the genes *TRI6*, *TRI10*, *pde1* (phosphodiesterase 1), *pde2* (phosphodiesterase 2), both involved in the degradation of cAMP, and the double mutants *ΔTRI6Δpde2* and *ΔTRI10Δpde2* were generated. The production of deoxynivalenol was observed to increase in response to the concentration of exogenous cAMP in the native strain; Likewise, an increase in the relative expression of the genes TRI5, TRI6, TRI10, TRI12, and TRI101 was identified in the presence of exogenous cAMP. When using phosphodiesterase inhibitors (IBMX and caffeine), there is an increase in the production of deoxynivalenol. However, when evaluating the *Δpde1* and *Δpde2* mutants, there is only an increase in deoxynivalenol production in the *Δpde2* mutant, which when subsequently complemented with the PDE2-GFP construct, recovers the wild phenotype. Based on these results, it was concluded that although phosphodiesterase 1 (PDE1) and phosphodiesterase 2 (PDE2) have overlapping functions, the regulation of deoxynivalenol synthesis depends mainly on PDE2.

By taking into account the mechanism of recognizing different carbohydrates by fungi, it is known that cAMP plays a key role in the signaling cascade (Dilks et al. 2019; Van Ende et al. 2019). For this reason, it is possible that the differential production of deoxynivalenol, observed in the work of Jiao et al. (2008), when using glucose or sucrose in the cultures in *F. graminearum*, depends on the levels of cAMP. However, the relationship between cAMP levels and deoxynivalenol synthesis is still unclear because Jiao et al. did not observe changes in the relative expression levels of *TRI6* and *TRI10*, while Jiang et al. show that there are significant differences regarding the relative expression levels of both genes in response to cAMP.

Although the role that CreA/CRE1 plays in the regulation of terpenoid synthesis by the carbon source remains debatable, it is not ruled out that this protein regulates the synthesis of some terpenoids; as has been observed with other types of secondary metabolites, either repressing as in the case of penicillins or inducing as in the case of some polyketides (Lind et al. 2016). In addition, the levels of cAMP may be determinant in the response of the differential synthesis of terpenoids due to the use of different carbon sources (Jiang et al. 2016). It must also be taken into account that the type of carbon source can affect the generation of energy in the cell; for this reason, it is also likely that the energy balance is affected, particularly affecting the activity of tailoring enzymes (specifically CYP450 monooxygenases).

## Regulation by the availability of cofactors

The last point of regulation in synthesizing secondary metabolites is at the level of enzymatic activity (Quin et al. 2015; Rodríguez-Concepción 2006; Sellés-Vidal et al. 2018). It has been observed that the cycling mechanism carried out by sesquiterpene synthases is affected by the concentration of divalent metals. By incubating homologues of Δ6-protoiludene synthase in vitro in the presence of Mg^2+^, Δ6-protoiludene is mainly produced. Still, by using Ca^2+^ as a metal cofactor, the production profile was changed, with β-elemene being the majority product (Quin et al. 2015). This is very interesting because this evidence suggests that the sesquiterpene synthase products can be directed by the type of divalent ions in the medium.

As mentioned above, remodeling enzymes diversify terpenoid compounds from a common base structure. Likewise, CYPs and oxidoreductases are the main enzymes in modifying the terpene skeleton. Therefore, the regulation of the activity of these enzymes must impact the formation of certain types of terpenoids. In anabolic pathways, the concentration of the NADPH cofactor is decisive for carrying out the function of many enzymes involved in the biosynthesis of various secondary metabolites. In addition, NADPH is used for the assimilation of some carbon sources, where oxidoreductases or CYP are involved (Sellés-Vidal et al. 2018). In the case of secondary metabolism, it has been observed that the production of some toxins such as fumonisins are affected when the NADP^+^/NADPH ratio increases in *Aspergillus niger*; because the formation of tricarbalilic esters depends on a dehydrogenase, orthologous of Fum7, dependent on NADPH. When supplementing the culture medium with different carbon sources: maltose, xylose, lactate, and pyruvate, it was observed that only in the case of lactate and pyruvate, which can increase the synthesis of NADH, there was an increase in the synthesis of fumonisins, without reach the levels reached when there is NADPH (Sørensen et al. 2009). It is known that many enzymes that use NADPH have evolved a particular cofactor binding site, for which the change in the NADP^+^/NADPH ratio toward the accumulation of the oxidized cofactor affects the capacity of oxidases and CYPs, being inhibited thus its function (Sellés-Vidal et al. 2018). However, there are also cases of oxidoreductases and CYPs that can use NADH as an additional cofactor to NADPH. Still, although its activity is not completely inhibited, it is significantly reduced, as in the case of CYP monooxygenases (Sellés-Vidal et al. 2018). This shows that the availability of the cofactors that can regulate the activity of the oxidoreductases and CYPs. However, it is still necessary to carry out studies focused on the regulation of the activity of these enzymes in secondary metabolism.

## Concluding remarks

Terpenoids are a group of compounds of great importance due to their biological properties; for this reason, knowing the synthesis and regulation process of these compounds is of utmost importance for biotechnological applications. Due to the diversity of terpene cyclases and their promiscuity, the most abundant terpenoids in fungi are the sesquiterpenoids. Likewise, with the help of the complete genomes of various fungi, the prediction of terpenoids in these organisms has been achieved. However, it is necessary to know under what conditions the terpenoids of interest can be produced since due to the various types of regulation, such as presenting different stages of development (mycelium, primordium, fruiting body), they can be a determining factor for the obtaining the compounds.

Within the mechanisms of regulation of the synthesis of terpenoids in fungi, the influence of various stressors such as light intensity, temperature, source of nutrients, pH and the presence of reactive oxygen species; influence the transcription of terpene synthases and consequently the production of terpenoids. However, applying the knowledge of how stressors affect the synthesis of terpenoids, the production of these compounds can be directed just by modifying fungi culture conditions. This will be extremely useful, particularly with the cultivation of basidiomycetes; since these are organisms difficult to manipulate genetically and consequently obtaining mutants for the overproduction of a compound is not very feasible and, with the use of these culture strategies, the synthesis of terpenoids of interest can be favored.

## Data Availability

The authors confirm that all relevant data are included in this article.

## Abbreviations

OSMAC: One Strain Many Compounds
IPP: Isopentenyl diphosphate
DMAPP: Dimethylallyl diphosphate
GPP: Geranyl diphosphate
FPP: Farnesyl diphosphate
GGPP: Geranylgeranyl diphosphate
GFPP: Geranylfarnesyl diphosphate
MEV: Mevalonate
MEP: 2-C-methyl-D-erythritol 4-phosphate
IPPS: Isoprenyl diphosphate synthases
TC: Terpene cyclase
NPP: Nerolidyl diphosphate
PTTS: Prenyltransferases-terpene syntases
UV: Ultraviolet
CYP: Cytochromes P450
HMG-CoA: 3-Hydroxy-3-methylglutaryl-CoA
SREBP: Sterol regulatory element-binding protein
SCAP: SREBP cleavage-activating protein
INSIG: Insulin-induced genes
ROS: Reactive oxygen species
CAMP: Cyclic adenosine monophosphate

## References

[CR1] Agger S, Lopez-Gallego F, Schmidt-Dannert C (2009). Diversity of sesquiterpene synthases in the basidiomycete *Coprinus cinereus*. Mol Microbiol.

[CR2] Akihisa T, Nakamura Y, Tagata M, Tokuda H, Yasukawa K, Uchiyama E, Suzuki T, Kimura Y (2007). Anti-inflammatory and anti-tumor-promoting effects of triterpene acids and sterols from the fungus *Ganoderma lucidum*. Chem Biodivers.

[CR3] Avalos J, Carmen Limón M (2015). Biological roles of fungal carotenoids. Curr Genet.

[CR4] Baby S, Johnson AJ, Govindan B (2015). Secondary metabolites from *Ganoderma*. Phytochem.

[CR5] Banerjee A, Sharkey TD (2013). Methylerythritol 4-phosphate (MEP) pathway metabolic regulation. Nat Prod Rep.

[CR6] Borgognone A, Castanera R, Morselli M, López-Varas L, Rubbi L, Pisabarro AG, Pellegrini M, Ramírez L (2018). Transposon-associated epigenetic silencing during *Pleurotus ostreatus* life cycle. DNA Res.

[CR7] Bryant JM, Bouchard M, Haque A (2017). Anticancer activity of ganoderic acid DM: current status and future perspective. J Clin Cell Immunol.

[CR8] Cale JA, Collignon RM, Klutsch JG, Kanekar SS, Hussain A, Erbilgin N (2016). Fungal volatiles can act as carbon sources and semiochemicals to mediate interspecific interactions among bark beetle-associated fungal symbionts. PLoS ONE.

[CR9] Calvo AM, Wilson RA, Bok JW, Keller NP (2002). Relationship between secondary metabolism and fungal development. Microbiol Mol Biol Rev.

[CR10] Caruthers JM, Kang I, Rynkiewicz MJ, Cane DE, Christianson DW (2000). Crystal structure determination of aristolochene synthase from the blue cheese mold *Penicillium roqueforti*. J Biol Chem.

[CR11] Chen S, Xu J, Liu C, Zhu Y, Nelson DR, Zhou S, Li C, Wang L, Guo X, Sun Y, Luo H, Li Y, Song J, Henrissat B, Levasseur A, Qian J, Li J, Luo X, Shi L, He L, Xiang L, Xu X, Niu Y, Li Q, Han MV, Yan H, Zhang J, Chen H, Lv A, Wang Z, Liu M, Schwartz DC, Sun C (2012). Genome sequence of the model medicinal mushroom *Ganoderma lucidum*. Nat Commun.

[CR12] Chen M, Harris GG, Pemberton TA, Christianson DW (2016). Multi-domain terpenoid cyclase architecture and prospects for proximity in bifunctional catalysis. Curr Opin Struct Biol.

[CR13] Chen J, Zeng X, Yang YL, Xing YM, Zhang Q, Li JM, Ma K, Liu HW, Guo SX (2017). Genomic and transcriptomic analyses reveal differential regulation of diverse terpenoid and polyketides secondary metabolites in *Hericium erinaceus*. Sci Rep.

[CR14] Chi B, Wang S, Bi S, Qin W, Wu D, Luo Z, Gui S, Wang D, Yin X, Wang F (2018). Effects of ganoderic acid A on lipopolysaccharide-induced proinflammatory cytokine release from primary mouse microglia cultures. Exp Ther Med.

[CR15] Choi S, Nguyen VT, Tae N, Lee S, Ryoo S, Min BS, Lee JH (2014). Anti-inflammatory and heme oxygenase-1 inducing activities of lanostane triterpenes isolated from mushroom *Ganoderma lucidum* in RAW264.7 cells. Toxicol App Pharmacol.

[CR16] Christianson DW (2008). Unearthing the roots of the terpenome. Curr Opin Chem Biol.

[CR17] Christianson DW (2017). Structural and chemical biology of terpenoid cyclases. Chem Rev.

[CR18] Citron CA, Wickel SM, Schulz B, Draeger S, Dickschat JS (2012). A Diels–Alder/retro-Diels–Alder approach for the enantioselective synthesis of microbial butenolides. Eur J Org Chem.

[CR19] Cui ML, Yang HY, He GQ (2015). Submerged fermentation production and characterization of intracellular triterpenoids from *Ganoderma lucidum* using HPLC-ESI-MS. J Zhejiang Univ Sci B.

[CR20] Degenhardt J, Köllner TG, Gershenzon J (2009). Monoterpene and sesquiterpene synthases and the origin of terpene skeletal diversity in plants. Phytochem.

[CR21] Dhingra S, Cramer RA (2017). Regulation of sterol biosynthesis in the human fungal pathogen *Aspergillus fumigatus*: opportunities for therapeutic development. Front Microbiol.

[CR22] Diamantopoulou P, Papanikolaou S, Komaitis M, Aggelis G, Philippoussis A (2014). Patterns of major metabolites biosynthesis by different mushroom fungi grown on glucose-based submerged cultures. Bioprocess Biosyst Eng.

[CR23] Dilks T, Halsey K, De Vos RP, Hammond-Kosack KE, Brown NA (2019). Non-canonical fungal G-protein coupled receptors promote *Fusarium* head blight on wheat. PLoS Pathog.

[CR24] Dubey A, Jeon J (2017). Epigenetic regulation of development and pathogenesis in fungal plant pathogens. Mol Plant Pathol.

[CR25] Dupont S, Lemetais G, Ferreira T, Cayot P, Gervais P, Beney L (2012). Ergosterol biosynthesis: a fungal pathway for life on land?. Evolution.

[CR26] El-Mekkaway SR, Meselhy M, Nakamura N, Tezuka Y, Hattori M, Kakiuchi N, Shimotohno K, Kawahata T, Otake T (1998). Anti-HIV-1 and anti-HIV-protease substances from *Ganoderma lucidum*. Phytochem.

[CR27] Espeso EA, Peñalva MA (1996). Three binding sites for the *Aspergillus nidulans* PacC zinc-finger transcription factor are necessary and sufficient for regulation by ambient pH of the isopenicillin N synthase gene promoter. J Biol Chem.

[CR28] Fang QH, Zhong JJ (2002). Submerged fermentation of higher fungus *Ganoderma lucidum* for production of valuable bioactive metabolites—ganoderic acid and polysaccharide. Biochem Eng J.

[CR29] Fukuda T, Kurihara Y, Kanamoto A, Tomoda H (2014). Terretonin G, a new sesterterpenoid antibiotic from marine-derived *Aspergillus* sp. OPMF00272. J Antibiot.

[CR30] Gershenzon J, Dudareva N (2007). The function of terpene natural products in the natural world. Nat Chem Biol.

[CR31] Golshayan D, Pascual M, Vogt B (2009). Mycophenolic acid formulations in adult renal transplantation—update on efficacy and tolerability. Ther Clin Risk Manag.

[CR32] González AG, León F, Rivera A, Padrón JI, González-Plata J, Zuluaga JC, Quintana J, Estévez F, Bermejo J (2002). New lanostanoids from the fungus *Ganoderma concinna*. J Nat Prod.

[CR33] Gutiérrez S, Marcos AT, Casqueiro J, Kosalková K, Fernández FJ, Velasco J, Martín JF (1999). Transcription of the *pcbAB, pc6C* and *penDE* genes of *Penicilliurn chrysogenum* AS-P-78 is repressed by glucose and the repression is not reversed by alkaline pHs. Microbiol.

[CR34] Han J, Chen Y, Bao L, Yang X, Liu D, Li S, Zhao F, Liu H (2013). Anti-inflammatory and cytotoxic cyathane diterpenoids from the medicinal fungus *Cyathus africanus*. Fitoterapia.

[CR35] Hartley AJ, de Mattos-Shipley K, Collins CM, Kilaru S, Foster GD, Bailey AM (2009). Investigating pleuromutilin-producing *Clitopilus* species and related basidiomycetes. FEMS Microbiol Lett.

[CR36] Hirota M, Morimura K, Shibata H (2002). Anti-inflammatory compounds from the bitter mushroom, *Sarcodon Scabrosus*. Biosci Biotechnol Biochem.

[CR37] Hou R, Jiang C, Zheng Q, Wang C, Xu JR (2015). The AreA transcription factor mediates the regulation of deoxynivalenol (DON) synthesis by ammonium and cyclic adenosine monophosphate (cAMP) signalling in *Fusarium graminearum*. Mol Plant Pathol.

[CR38] Hu Y, Ahmed S, Li J, Luo B, Gao Z, Zhang Q, Li X, Hu X (2017). Improved ganoderic acids production in *Ganoderma lucidum* by wood decaying components. Sci Rep.

[CR39] Huang W, Shang Y, Chen P, Cen K, Wang C (2015). Basic leucine zipper (bZIP) domain transcription factor MBZ1 regulates cell wall integrity, spore adherence, and virulence in *Metarhizium robertsii*. J Biol Chem.

[CR40] Ishikawa NK, Yamaji K, Miura IH, K, Fukushi Y, Takahashi K, Tahara S,  (2005). Production of enokipodins A, B, C, and D: a new group of antimicrobial metabolites from mycelial culture of *Flammulina velutipes*. Mycoscience.

[CR41] Jaenisch R, Bird A (2003). Epigenetic regulation of gene expression: how the genome integrates intrinsic and environmental signals. Nat Genet.

[CR42] Jansen BJM, de Groot A (2004). Occurrence, biological activity and synthesis of drimane sesquiterpenoids. Nat Prod Rep.

[CR43] Jiang C, Zhang C, Wu C, Sun P, Hou R, Liu H, Wang C, Xu JR (2016). TRI6 and TRI10 play different roles in the regulation of deoxynivalenol (DON) production by cAMP signalling in *Fusarium graminearum*. Environ Microbiol.

[CR44] Jiao F, Kawakami A, Nakajima T (2008). Effects of different carbon sources on trichothecene production and Tri gene expression by *Fusarium graminearum* in liquid culture. FEMS Microbiol Lett.

[CR45] Johnson BM, DeBose-Boyd RA (2018). Underlying mechanisms for sterol-induced ubiquitination and ER-associated degradation of HMG CoA reductase. Semin Cell Dev Biol.

[CR46] Jordá T, Puig S (2020). Regulation of ergosterol biosynthesis in *Saccharomyces cerevisiae*. Genes.

[CR47] Kawakami A, Nakajima T, Hirayae K (2014). Effects of carbon sources and amines on induction of trichothecene production by *Fusarium asiaticum* in liquid culture. FEMS Microbiol Lett.

[CR48] Kenne GJ, Gummadidala PM, Omebeyinje MH, Mondal AM, Bett DK, McFadden S, Bromfield S, Banaszek N, Velez-Martinez M, Mitra C, Mikell I, Chatterjee S, Wee J, Chanda A (2018). Activation of aflatoxin biosynthesis alleviates total ROS in *Aspergillus parasiticus*. Toxins (Basel).

[CR49] Kimura M, Kushiro T, Shibuya M, Ebizuka Y, Abe I (2009). Protostadienol synthase from *Aspergillus fumigatus*: functional conversion into lanosterol synthase. Biochem Biophys Res Commun.

[CR50] Kuhnert E, Li Y, Lan N, Yue Q, Chen L, Cox RJ, An Z, Yokoyama K, Bills GF (2018). Enfumafungin synthase represents a novel lineage of fungal triterpene cyclases. Environ Microbiol.

[CR51] Kuzuyama T, Seto H (2012). Two distinct pathways for essential metabolic precursors for isoprenoid biosynthesis. Proc Jpn Acad Ser B.

[CR52] Langenheim JH (1994). Higher plant terpenoids: a phytocentric overview of their ecological roles. J Chem Ecol.

[CR53] Lauterbach L, Wang T, Stadler M, Dickschat JS (2019). Volatiles from the ascomycete *Daldinia cf*. *childiae* (Hypoxylaceae), originating from China. Med Chem Comm.

[CR54] Li F, Wang Y, Wang X, Li J, Cui H, Niu M (2012). Ganoderic acids suppress growth and angiogénesis by modulating the NF-κB signaling pathway in breast cancer cells. Int J Clin Pharmacol Ther.

[CR55] Li P, Deng YP, Wei XX, Xu JH (2013). Triterpenoids from *Ganoderma lucidum* and their cytotoxic activities. Nat Prod Res.

[CR56] Lim FY, Sanchez JF, Wang CC, Keller NP (2012). Toward awakening cryptic secondary metabolite gene clusters in filamentous fungi. Methods Enzymol.

[CR57] Lin L, Xu J (2020). Fungal pigments and their roles associated with human health. J Fungi (basel).

[CR58] Lind AL, Smith TD, Saterlee T, Calvo AM, Rokas A (2016). Regulation of secondary metabolism by the velvet complex is temperature-responsive in *Aspergillus*. G3 (Bethesda).

[CR59] Liu XT, Winkler AL, Schwan WR, Volk TJ, Rott MA, Monte A (2010). Antibacterial compounds from mushrooms I: a lanostane-type triterpene and prenylphenol derivatives from *Jahnoporus hirtus* and *Albatrellus flettii* and their activities against *Bacillus cereus* and *Enterococcus faecalis*. Planta Med.

[CR60] Lopez-Gallego F, Agger SA, Abate-Pella D, Distefano MD, Schmidt-Dannert C (2010). Sesquiterpene synthases Cop4 and Cop6 from *Coprinus cinereus*: catalytic promiscuity and cyclization of farnesyl pyrophosphate geometric isomers. ChemBioChem.

[CR61] Macías-Rubalcava ML, Hernández-Bautista BE, Oropeza F, Duarte G, González MC, Glenn AE, Hanlin RT, Anaya AL (2010). Allelochemical effects of volatile compounds and organic extracts from *Muscodor yucatanensis*, a tropical endophytic fungus from *Bursera simaruba*. J Chem Ecol.

[CR62] Martin DM, Gershenzon J, Bohlmann J (2003). Induction of volatile terpene biosynthesis and diurnal emission by methyl jasmonate in foliage of Norway spruce. Plant Physiol.

[CR63] Mazur X, Becker U, Anke T, Sterner O (1996). Two new bioactive diterpenes from *Lepista sordida*. Phytochem.

[CR64] Mihlan M, Homann V, Liu TW, Tudzynski B (2003). AREA directly mediates nitrogen regulation of gibberellin biosynthesis in *Gibberella fujikuroi*, but its activity is not affected by NMR. Mol Microbiol.

[CR65] Mitsuguchi H, Seshime Y, Fujii I, Shibuya M, Ebizuka Y, Kushiro T (2009). Biosynthesis of steroidal antibiotic fusidanes: functional analysis of oxidosqualene cyclase and subsequent tailoring enzymes from *Aspergillus fumigatus*. J Am Chem Soc.

[CR66] Mitsuhashi T, Abe I (2018). Chimeric terpene synthases possessing both terpene cyclization and prenyltransfer activities. ChemBioChem.

[CR67] Miyanga A (2017). Structure and function of polyketide biosynthetic enzymes: various strategies for production of structurally diverse polyketides. Biosci Biotechnol Biochem.

[CR68] Mothana RA, Jansen R, Jülich WD, Lindequist U (2000). Ganomycins A and B, new antimicrobial farnesyl hydroquinones from the basidiomycete *Ganoderma pfeifferi*. J Nat Prod.

[CR69] Nazari L, Manstretta V, Rossi V (2016). A non-linear model for temperature-dependent sporulation and T-2 and HT-2 production of *Fusarium langsethiae* and *Fusarium sporotrichioides*. Fungal Biol.

[CR70] Nelson DL, Cuchillo-Foix CM, Lehninger AL, Cox MM (2005). Lehninger: Principios de Bioquímica.

[CR71] Ou YX, Li YY, Qian XM, Shen YM (2012). Guanacastane-type diterpenoids from *Coprinus radians*. Phytochemistry.

[CR72] Palenzuela M, Sánchez-Roa D, Damián J, Sessini V, Mosquera MEG, Pérez PJ (2021). Chapter two—polymerization of terpenes and terpenoids using metal catalysts. Advances in organometallic chemistry.

[CR73] Peters RJ (2010). Two rings in them all: the labdane-related diterpenoids. Nat Prod Rep.

[CR74] Portnoy T, Margeot A, Linke R, Atanasova L, Fekete E, Sándor E, Hartl L, Karaffa L, Druzhinina IS, Seiboth B, Le Crom S, Kubicek CP (2011). The CRE1 carbon catabolite repressor of the fungus *Trichoderma reesei*: a master regulator of carbon assimilation. BMC Genomics.

[CR75] Pruss S, Fetzner R, Seither K, Herr A, Pfeiffer E, Metzler M, Lawrence CB, Fischer R (2014). Role of the *Alternaria alternata* blue-light receptor LreA (white-collar 1) in spore formation and secondary metabolism. Appl Environ Microbiol.

[CR76] Quin MB, Flyn CM, Schmidt-Dannert C (2014). Traversing the fungal terpenome. Nat Prod Rep.

[CR77] Quin MB, Michel SN, Schmidt-Dannert C (2015). Moonlighting metals: insights into regulation of cyclization pathways in fungal Δ(6)-protoilludene sesquiterpene synthases. ChemBioChem.

[CR78] Rapior S, Breheret S, Talou T, Pelissier Y, Milhau M, Bessiere JM (1998). Volatile components of fresh *Agrocybe aegerita* and *Tricholoma Sulfureum*. Cryptogamie Mycol.

[CR79] Rinkel J, Rabe P, Horts L, Dickschat JS (2016). A detailed view on 1,8-cineol biosynthesis by *Streptomyces clavuligerus*. Beilstein J Org Chem.

[CR80] Rodríguez-Concepción M (2006). Early steps in isoprenoid biosynthesis: multilevel regulation of the supply of common precursors in plant cells. Phytochem Rev.

[CR81] Rodríguez-Concepción M, Boronat A (2002). Elucidation of the methylerythritol phosphate pathway for isoprenoid biosynthesis in bacteria and plastids. A metabolic milestone achieved through genomics. Plant Physiol.

[CR82] Schmidt-Dannert C (2015). Biosynthesis of terpenoid natural products in fungi. Adv Biochem Eng Biotechnol.

[CR83] Schrader J, Bohlmann J (2015). Biotechnology of isoprenoids.

[CR84] Sellés-Vidal L, Kelly CL, Mordaka PM, Heap JT (2018). Review of NAD(P)H-dependent oxidoreductases: properties, engineering and application. Biochim Biophys Acta Proteins Proteom.

[CR85] Seo HW, Hung TM, Na M, Jung HJ, Kim JC, Choi JS, Kim JH, Lee HK, Lee I, Bae K, Hattori M, Min BS (2009). Steroids and triterpenes from the fruit bodies of *Ganoderma lucidum* and their anti-complement activity. Arch Pharm Res.

[CR86] Shaw JJ, Berbasova T, Sasaki T, Jefferson-George K, Spakowicz DJ, Dunican BF, Portero CE, Narváez-Trujillo A, Strobel SA (2015). Identification of a fungal 1,8-cineole synthase from *Hypoxylon* sp. with specificity determinants in common with the plant synthases. J Biol Chem.

[CR87] Shibata H, Irie A, Morita Y (1998). New antibacterial diterpenoids from the *Sarcodon scabrosus* fungus. Biosci Biotechnol Biochem.

[CR88] Sørensen LM, Lametsch R, Andersen MR, Nielsen PV, Frisvad JC (2009). Proteome analysis of *Aspergillus niger*: lactate added in starch-containing medium can increase production of the mycotoxin fumonisin B2 by modifying acetyl-CoA metabolism. BMC Microbiol.

[CR89] Strauss J, Reyes-Dominguez Y (2011). Regulation of secondary metabolism by chromatin structure and epigenetic codes. Fungal Genet Biol.

[CR90] Sun LP, Li L, Goldstein JL, Brown MS (2005). Insig required for sterol-mediated inhibition of Scap/SREBP binding to COPII proteins *in vitro*. J Biol Chem.

[CR91] Szkopińska A, Rytka J, Karst F, Palamarczyk G (1993). The deficiency of sterol biosynthesis in *Saccharomyces cerevisiae* affects the synthesis of glycosyl derivatives of dolichyl phosphates. FEMS Microbiol Lett.

[CR92] Tabuchi A, Fukushima-Sakuno E, Osaki-Oka K, Futamura Y, Motoyama T, Osada H, Ishikawa NK, Nagasawa E, Tokimoto K (2020). Productivity and bioactivity of enokipodins A-D of *Flammulina rossica* and *Flammulina velutipes*. Biosci Biotechnol Biochem.

[CR93] Tagami K, Liu C, Minami A, Noike M, Isaka T, Fueki S, Shichijo Y, Toshima H, Gomi K, Dairi T, Oikawa H (2013). Reconstitution of biosynthetic machinery for indole-diterpene paxilline in *Aspergillus oryzae*. J Am Chem Soc.

[CR94] Tang YA, Zhong JJ (2002). Fed-batch fermentation of *Ganoderma lucidum* for hyperproduction of polysaccharide and ganoderic acid. Enzyme Microb Technol.

[CR95] Tello M, Kuzuyama T, Heide L, Noel JP, Richard SB (2008). The ABBA family of aromatic prenyltransferases: broadening natural product diversity. Cell Mol Life Sci.

[CR96] Tholl D (2015). Biosynthesis and biological functions of terpenoids in plants. Adv Biochem Eng Biotechnol.

[CR97] Valdivia C, Kettering M, Anke H, Thines E, Sterner O (2005). Diterpenoids from *Coprinus heptemerus*. Tetrahedron.

[CR98] Van Ende M, Wijnants S, Van Dijck P (2019). Sugar sensing and signaling in *Candida albicans* and *Candida glabrata*. Front Microbiol.

[CR99] Vonk PJ, Ohm RA (2021). H3K4me2 ChIP-Seq reveals the epigenetic landscape during mushroom formation and novel developmental regulators of *Schizophyllum commune*. Sci Rep.

[CR100] Wang G, Zhao J, Liu JW, Huang Y, Zhong JJ, Tang W (2007). Enhancement of IL-2 and IFN-γ expression and NK cells activity involved in the anti-tumor effect of ganoderic acid Me *in vivo*. Int Immunopharmacol.

[CR101] Wang K, Bao L, Xiong W, Ma K, Han J, Wang W, Yin W, Liu H (2015). Lanostane triterpenes from the tibetan medicinal mushroom *Ganoderma leucocontextum* and their inhibitory effects on HMG-CoA reductase and α-glucosidase. J Nat Prod.

[CR102] Wang C, Schröder MS, Hammel S, Butler G (2016). Using RNA-seq for analysis of differential gene expression in fungal species. Methods Mol Biol.

[CR103] Wawrzyn GT, Quin MB, Choudhary S, López-Gallego F, Schmidt-Dannert C (2012). Draft genome of *Omphalotus olearius* provides a predictive framework for sesquiterpenoid natural product biosynthesis in Basidiomycota. Chem Biol.

[CR104] Weikl F, Ghirardo A, Schnitzler JP, Pritsch K (2016). Sesquiterpene emissions from *Alternaria alternata* and *Fusarium oxysporum*: effects of age, nutrient availability, and co-cultivation. Sci Rep.

[CR105] Yan Y, Li M, Zhang X, Kong W, Bendahmane M, Bao M, Fu X (2022). Tissue-specific expression of the terpene synthase family genes in *Rosa chinensis* and effect of abiotic stress conditions. Genes.

[CR106] Yang T, Espenshade PJ, Wright ME, Yabe D, Gong Y, Aebersold R, Goldstein JL, Brown MS (2002). Crucial step in cholesterol homeostasis: sterols promote binding of SCAP to INSIG-1, a membrane protein that facilitates retention of SREBPs in ER. Cell.

[CR107] Yang HG, Zhao H, Li JJ, Chen SM, Mou LM, Zou J, Chen GD, Qin SY, Wang CX, Hu D, Yao XS, Gao H (2017). Phyllomeroterpenoids A-C, multi-biosynthetic pathway derived meroterpenoids from the TCM endophytic fungus *Phyllosticta* sp. and their antimicrobial activities. Sci Rep.

[CR108] Yin H, Han S, Chen Y, Wang Y, Li D, Zhu Q (2020). T-2 toxin induces oxidative stress, apoptosis and cytoprotective autophagy in chicken hepatocytes. Toxins..

[CR109] Ying YM, Shan WG, Zhang LW, Zhan ZJ (2013). Ceriponols A-K, tremulane sesquitepenes from *Ceriporia lacerata* HS-ZJUT-C13A, a fungal endophyte of *Huperzia serrata*. Phytochem.

[CR110] Yue QX, Song XY, Ma C, Feng LX, Guan SH, Wu WY, Yang M, Jiang BH, Liu X, Cui YJ, Guo DA (2010). Effects of triterpenes from *Ganoderma lucidum* on protein expression profile of HeLa cells. Phytomedicine.

[CR111] Zhang X, Hou X, Xu D, Xue M, Zhang J, Wang J, Yang Y, Lai D, Zhou L (2023). Effects of carbon, nitrogen, ambient pH and light on mycelial growth, sporulation, sorbicillinoid biosynthesis and related gene expression in *Ustilaginoidea virens*. J Fungi.

